# Additive effects of booster mRNA vaccination and SARS-CoV-2 Omicron infection on T cell immunity across immunocompromised states

**DOI:** 10.1126/scitranslmed.adg9452

**Published:** 2023-07-12

**Authors:** Thomas R. Müller, Takuya Sekine, Darya Trubach, Julia Niessl, Puran Chen, Peter Bergman, Ola Blennow, Lotta Hansson, Stephan Mielke, Piotr Nowak, Jan Vesterbacka, Mira Akber, Anna Olofsson, Susana Patricia Amaya Hernandez, Yu Gao, Curtis Cai, Gunnar Söderdahl, C. I. Edvard Smith, Anders Österborg, Karin Loré, Margaret Sällberg Chen, Per Ljungman, Hans-Gustaf Ljunggren, Annika C. Karlsson, Sunil Kumar Saini, Soo Aleman, Marcus Buggert

**Affiliations:** 1 Department of Medicine Huddinge, Center for Infectious Medicine, Karolinska Institutet, Stockholm, Sweden; 2 Department of Health Technology, Section of Experimental and Translational Immunology, Technical University of Denmark, Kongens Lyngby, Denmark; 3 Department of Infectious Diseases, Karolinska University Hospital, Stockholm, Sweden; 4 Department of Laboratory Medicine, Clinical Immunology, Karolinska Institutet, Stockholm, Sweden; 5 Department of Clinical Immunology and Transfusion Medicine, Karolinska University Hospital, Stockholm, Sweden; 6 Department of Transplantation, Karolinska University Hospital, Stockholm, Sweden; 7 Department of Medicine Huddinge, Infectious Diseases, Karolinska Institutet, Stockholm, Sweden; 8 Department of Hematology, Karolinska University Hospital, Stockholm, Sweden; 9 Department of Oncology-Pathology, Karolinska Institutet, Stockholm, Sweden; 10 Department of Laboratory Medicine, Biomolecular and Cellular Medicine, Karolinska Institutet, Stockholm, Sweden; 11 Department of Cellular Therapy and Allogeneic Stem Cell Transplantation (CAST), Karolinska Comprehensive Cancer Center, Karolinska University Hospital Huddinge, Stockholm, Sweden; 12 Laboratory for Molecular Infection Medicine Sweden MIMS, Umeå University, Sweden; 13 Division of Clinical Microbiology, Department of Laboratory Medicine, Karolinska Institutet, Stockholm, Sweden; 14 Department of Clinical Science, Intervention and Technology, Karolinska Institutet, Stockholm, Sweden; 15 Department of Medicine Solna, Karolinska Institutet, Karolinska University Hospital, Stockholm, Sweden; 16 Department of Dental Medicine, Karolinska Institutet, Stockholm, Sweden; 17 Department of Medicine Huddinge, Hematology, Karolinska Institutet, Stockholm; 18 Karolinska University Laboratory, Clinical Microbiology, Karolinska University Hospital, Stockholm, Sweden

## Abstract

Suboptimal immunity to SARS-CoV-2 mRNA vaccination has frequently been observed in individuals with various immunodeficiencies. Given the increased antibody evasion properties of emerging SARS-CoV-2 subvariants, it is necessary to assess whether other components of adaptive immunity generate resilient and protective responses against infection. We assessed T cell responses in 279 individuals, covering five different immunodeficiencies and healthy controls, before and after booster mRNA vaccination, as well as following Omicron infection in a subset of patients. We observed robust and persistent Omicron-reactive T cell responses that increased markedly upon booster vaccination and correlated directly with antibody titers across all patient groups. Poor vaccination responsiveness in immunocompromised or elderly individuals was effectively counteracted by the administration of additional vaccine doses. Functionally, Omicron-reactive-T cell responses exhibited a pronounced cytotoxic profile and signs of longevity, characterized by CD45RA^+^ effector memory subpopulations with stem-cell-like properties and increased proliferative capacity. Regardless of underlying immunodeficiency, booster-vaccinated and Omicron-infected individuals appeared protected against severe disease and exhibited enhanced and diversified T cell responses against conserved and Omicron-specific epitopes. Our findings indicate that T cells retain the ability to generate highly functional responses against newly emerging variants, even following repeated antigen exposure and a robust immunological imprint from ancestral SARS-CoV-2 mRNA vaccination.

## Introduction

In late 2021, Omicron (B.1.1.529) and its subvariants began to evolutionarily outcompete earlier SARS-CoV-2 variants. A plethora of mutations in the spike glycoprotein largely conferred evasion from antibody responses induced by the ancestral Wuhan strain (Wu-Hu.1)-based vaccine (1, 2). Consequently, protection from infection was dramatically decreased, facilitating the global spread of Omicron (3). Fortunately, protection from severe disease has remained high among vaccinated or convalescent individuals (4, 5), due in part to maintained cross-reactive immunity by T cell responses, which have remained critical for SARS-CoV-2 immunity (6, 7). Indeed, robust Omicron-reactive T cell responses were observed in samples of fully vaccinated or healthy convalescent individuals collected before the emergence of Omicron (8–10), indicating only marginal escape from T cell-mediated immunity. In contrast to the antibody response, T cells recognize intracellularly processed antigens in the context of various HLA molecules, which generates large diversity of the antigenic and TCR repertoire, ultimately hampering the evolution of escape mutations. However, it is still under debate whether antigenic sin, an effect that describes poor memory recall responses to mutated versions of the original antigen (11–13), might dampen T cell immunity to newly emerging variants upon initial vaccination or infection with the ancestral strain.

COVID-19 risk groups, such as the elderly with co-morbidities or immunocompromised individuals, are known to mount overall weaker responses after vaccination (14) and can display restricted TCR repertoire diversity (15, 16). Impaired SARS-CoV-2 mRNA vaccine-induced cell-mediated immunity was observed in immunocompromised individuals (17–19), leaving them at higher risk of infection and severe disease even after two vaccine doses (20, 21). Administration of an additional booster vaccine dose does increase protection from SARS-CoV-2 infection (22), leading to the recommendation that COVID-19 risk groups receive additional booster shots in many countries, including Sweden. However, it has remained unclear to which extent these individuals would benefit from additional vaccine doses, particularly regarding protection against the newly emerged Omicron variant.

Here, we investigated Wu-Hu.1 and Omicron spike-reactive T cell responses before and after booster vaccination and with or without an additional Omicron infection in healthy donors and across various immunocompromised states, including primary immunodeficiency (PID), human immunodeficiency virus type 1 (HIV) infection, post hematopoietic stem cell transplantation (HSCT), post solid organ transplantation (SOT), and chronic lymphocytic leukemia (CLL). To assess the magnitude, phenotype, and function of Wu-Hu.1 spike-specific versus Omicron spike cross-reactive responses, as well as Omicron spike-specific responses, we performed stimulations with peptide pools spanning the entire spike region of Wu-Hu.1 and Omicron B.1.1.529 or, respectively, only the mutated regions of the Omicron subvariant. Further, we performed epitope mapping for the dissection of T cell repertoire perturbations upon booster vaccination and additional Omicron infection to study potential antigenic sin effects.

Our findings highlight the importance of booster vaccination in immunocompromised individuals to sufficiently build up functional and long-living Omicron-reactive CD8^+^ T cell responses that likely contribute to protection from severe disease. Hybrid immunity through additional Omicron infection further ameliorates and diversifies SARS-CoV-2 T cell responses, including the formation of Omicron-specific T cell immunity, despite a solid immunological imprint derived from three to four ancestral-based vaccine doses.

## Results

### Booster with ancestral mRNA vaccine generates robust Omicron-reactive T cell immunity

**Fig. 1:**
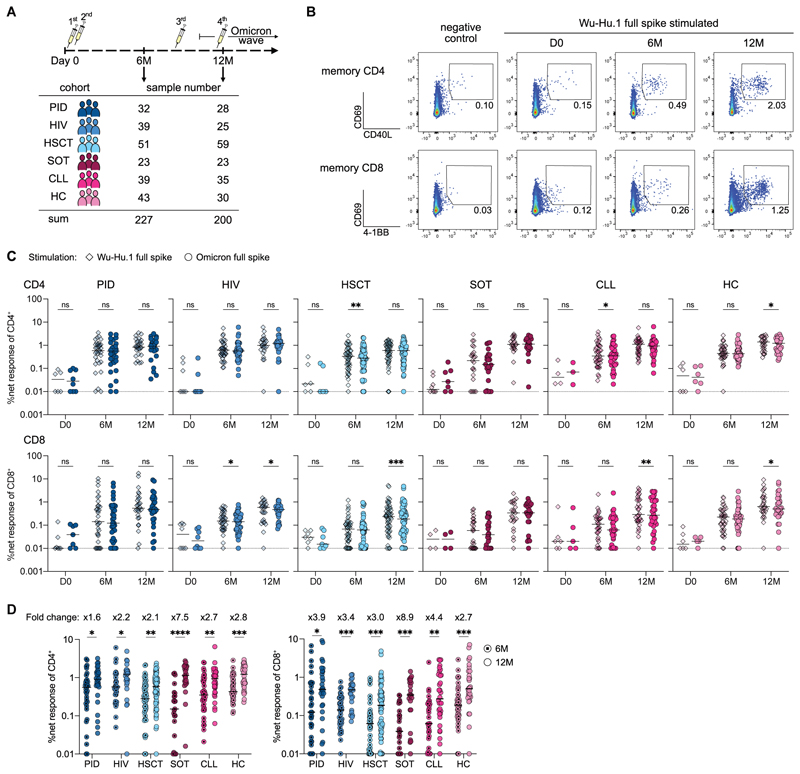
Booster with ancestral mRNA vaccine generates robust Omicron-reactive T cell responses across various immunocompromised states. **(A)** Schematic of the longitudinal study design involving six groups of healthy and immunocompromised patients. **(B)** Representative plots depicting upregulation of activation markers on memory CD4^+^ (CD69, CD40L) and memory CD8^+^ (CD69, 4-1BB) T cells upon stimulation with negative control or Wu-Hu.1 full spike peptide pool over time. **(C)** Net frequencies (background-subtracted using DMSO negative control) of T cell responses to Wu-Hu.1 full spike and Omicron full spike peptide pools over time across all patient groups. **(D)** Comparison of pre- and post-booster net frequencies of CD4 and CD8 Omicron-reactive T cell responses with indicated median fold changes. (A to D) PID, primary immunodeficiency; HIV, human immunodeficiency virus type 1; HSCT, hematopoietic stem-cell transplantation; SOT, solid organ transplantation; CLL, chronic lymphocytic leukemia; HC, healthy controls. (C, D) Each dot represents one donor and lines depict the median. (C) Wilcoxon matched-pairs signed rank test for Wu-Hu.1 versus Omicron comparison with Holm-Šidák posttest to correct for multiple comparisons. (D) Mann-Whitney test with Holm-Šidák posttest. ****p <0.0001, ^***^p <0.001, **p <0.01, *p <0.05.

To examine the impact of booster vaccination on Wu-Hu.1 and Omicron spike-reactive T cell immunity in healthy individuals as well as across various immunocompromised states, we performed longitudinal blood sampling of patients from the prospective open-label clinical trial COVAXID (EudraCT, no. 2021-000175-37) (23). All study participants had negative PCR tests at baseline, no previous documented COVID-19, and received three to four doses of the Wu-Hu.1-based mRNA vaccine. Peripheral blood mononuclear cells (PBMC) were collected before the first dose at day 0, before the third dose at month 6 (6M), and after booster vaccination at month 12 (12M). Concomitantly, a subset of individuals was infected during the Omicron wave, which occurred between the 6- and 12-month sampling time points (Fig. 1A). Baseline cohort characteristics, along with information on sampling times and the number of received vaccine doses, are described in Supplementary Table 1. We performed short-term *in vitro* stimulation of PBMCs with overlapping peptide pools spanning the whole spike region of either the ancestral Wuhan strain (Wu-Hu.1 full spike) or the Omicron B.1.1.529 (Omicron full spike) strain to longitudinally track memory CD4^+^ and CD8^+^ T cell responses via activation-induced marker (AIM) staining (Fig. 1B, see Suppl. Fig. 1A for gating strategy). Comparison of T cell responses reactive against either Wu-Hu.1 or Omicron spike revealed no or only marginal differences, highlighting robust T cell cross-recognition of the Omicron variant (Fig. 1C), as observed in healthy individuals (8–10). Comparison of T cell responses before (6M) and after booster vaccination (12M) revealed significantly increased Omicron spike-reactive CD4^+^ and CD8^+^ T cell responses (Fig. 1D). The gains in response magnitude were particularly notable in the CD8^+^ T cell compartment, as indicated by increased 12-month/6-month fold changes (Fig. 1D), resulting in slightly increased CD8/CD4-ratios upon booster vaccination (Suppl. Fig. 1B). To contextualize booster vaccination-induced T cell response increases, we compared these responses to those directly after full vaccination. T cell responses to SARS-CoV-2 infection and vaccination have been reported to decay over time (24, 25), so we calculated CD4^+^ T cell response decay from day 35 (two weeks after the second dose) to month 6 based on earlier data on the same cohort published by our group (18). CD4^+^ T cell decay rates did not differ significantly between patient groups (Suppl. Fig. 1C) and were used to extrapolate day 35 CD4^+^ T cell data based on the 6-month CD4^+^ T cell responses measured within this study. Across all patient groups, CD4^+^ T cell responses were larger at 12 months compared to day 35, particularly in SOT, CLL, and HC (Suppl. Fig. 1D). Since the timing of the vaccine doses and sampling were more variable at the 6-month and 12-month time points compared to earlier time points in this clinical trial, we investigated whether this might introduce bias. To this end, we measured correlations between the elapsed time since the second or last dose with 6-month and 12-month T cell responses, respectively. We did not observe any correlations, indicating that the differential time span between vaccination and sampling is not a confounding parameter in our analyses (Suppl. Fig. 1E). Collectively, we observed that additional booster doses significantly increased Wu-Hu.1 and Omicron spike-reactive T cell responses, particularly within the CD8^+^ T cell compartment, across all patient groups.

### Variable vaccine responsiveness can be counteracted by the number of doses

**Fig. 2:**
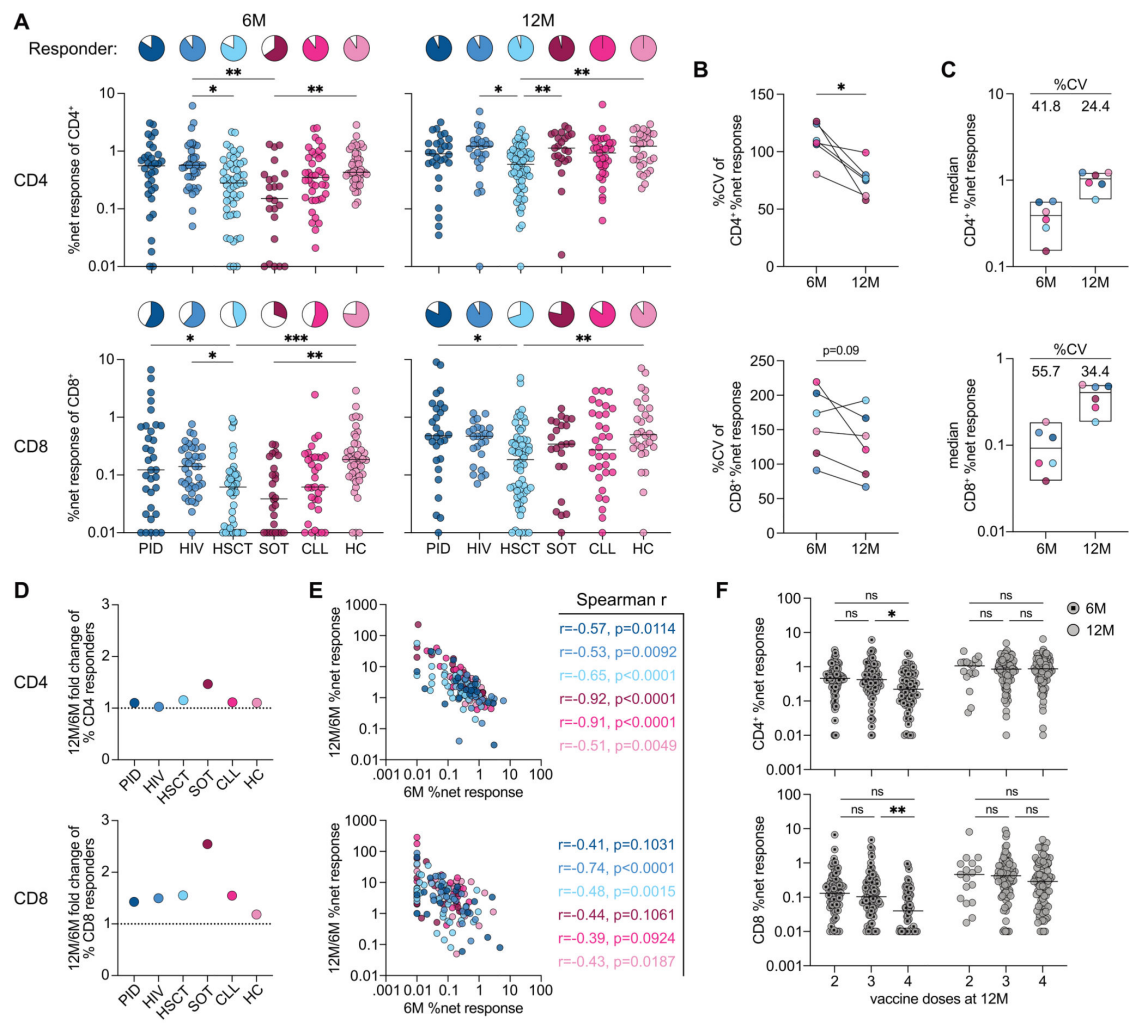
Individual variability of vaccine-induced Omicron-reactive T cell responses can be counteracted by number of doses. **(A)** Patient group comparison of net frequencies and responder frequencies (defined by response ≥ 2 x background) of T cell responses at 6M and 12M. **(B)** Coefficient of variation (CV) of T cell responses within individual patient groups at 6M and 12M. **(C)** Median T cell response sizes of individual patient groups at 6M and 12M and respective coefficient of variation between groups. **(D)** Change of mean CD4 and CD8 responder frequency between pre- and post-booster. **(E)** Correlation of pre-booster net frequencies with the ratio of net frequencies between pre- and post-booster with indicated Spearman correlation and p-values. **(F)** Comparison of pre- and post-booster T cell responses based on the number of received vaccine doses at 12M. Pooled data of entire cohort (see Suppl. Fig. 2A for patient group data). (A to F) T cell responses to Omicron full spike peptide pool. (A, E, F) Each dot represents one donor and lines depict the median. (C, D) Each dot represents the median of one patient group. (A) Kruskal Wallis test with Dunn’s posttest for patient group comparison. (B) Wilcoxon matched-pairs signed rank test. (F) Mixed-effects model with the Geisser-Greenhouse correction with Tukey’s multiple comparisons test. ^***^p <0.001, **p <0.01, *p <0.05.

Comparison of different patient groups revealed significantly lower Omicron-reactive T cell responses and responder frequencies in HSCT and SOT, particularly before booster vaccination (Fig. 2A). Differences in Omicron-reactive T cell responses within (Fig. 2B) and between patient groups (Fig. 2C) were more pronounced at 6 months, indicating more leveled response sizes after booster vaccination. The observation of both response variability before but also similar response magnitudes after booster vaccination prompted us to investigate which patient groups and individuals benefitted most from additional dose administrations. The SOT group experienced the highest increase in responders, while the lowest gains were observed in healthy controls (Fig. 2D).

To investigate the impact of the underlying disease on vaccination responsiveness, we analyzed T cell responses in patient subgroups and observed particularly low responses in patients with active immunosuppressive medication (e.g., transplant recipients or CLL patients receiving Ibrutinib or mycophenolate mofetil (MMF), Suppl. Fig. 2A). Again, Omicron-reactive T cell response magnitude differences between subgroups were less pronounced after booster vaccination. Looking at individual patients across all groups, we observed a negative correlation between Omicron-reactive T cell response magnitude before booster vaccination and response increase upon booster, both in the CD4^+^ and CD8^+^ T cell compartments (Fig. 2E). The slightly weaker correlations in the CD8^+^ T cell compartment can likely be attributed to generally weaker (or even non-detectable) responses at 6 months, resulting in higher variability in the data. Notably, the observed correlations indicate that poor responders after two vaccine doses particularly benefit from booster vaccination, regardless of their underlying disease. We further analyzed how age influences vaccination responsiveness in our cohort and observed a negative correlation that was more pronounced at the 6-month time point, as well as in healthy controls who do not present with any underlying disease (Suppl. Fig. 2B).

These data indicated that both disease (especially individuals on active immunosuppression) and advanced age negatively impact responsiveness to two vaccine doses. However, we also observed less variability in T cell responses at 12 months, suggesting that cell-mediated immunity may saturate upon booster vaccination. To investigate this more closely, we analyzed CD4^+^ and CD8^+^ Omicron-reactive T cell responses based on the number of received vaccine doses at the 12-month time point. Across all patient groups, individuals receiving the highest number of doses also exhibited the largest response increases (Suppl. Fig. 2C). Interestingly, we observed that individuals who received the most doses also had significantly lower T cell responses before booster dose administration, whereas response levels were similar afterward, irrespective of the number of received doses (Fig. 2F). Collectively, variable vaccination responsiveness due to disease and age was effectively counteracted through adaptation of the number of vaccine doses, resulting in similarly high T cell response levels across the entire cohort. Of note, the same findings apply to Wu-Hu.1-reactive vaccination responses, as indicated through almost identical response sizes shown in Fig. 1C.

### Vaccine-induced T cell responses are synchronized with antibody titers and display an inflammatory immune signature

**Fig. 3:**
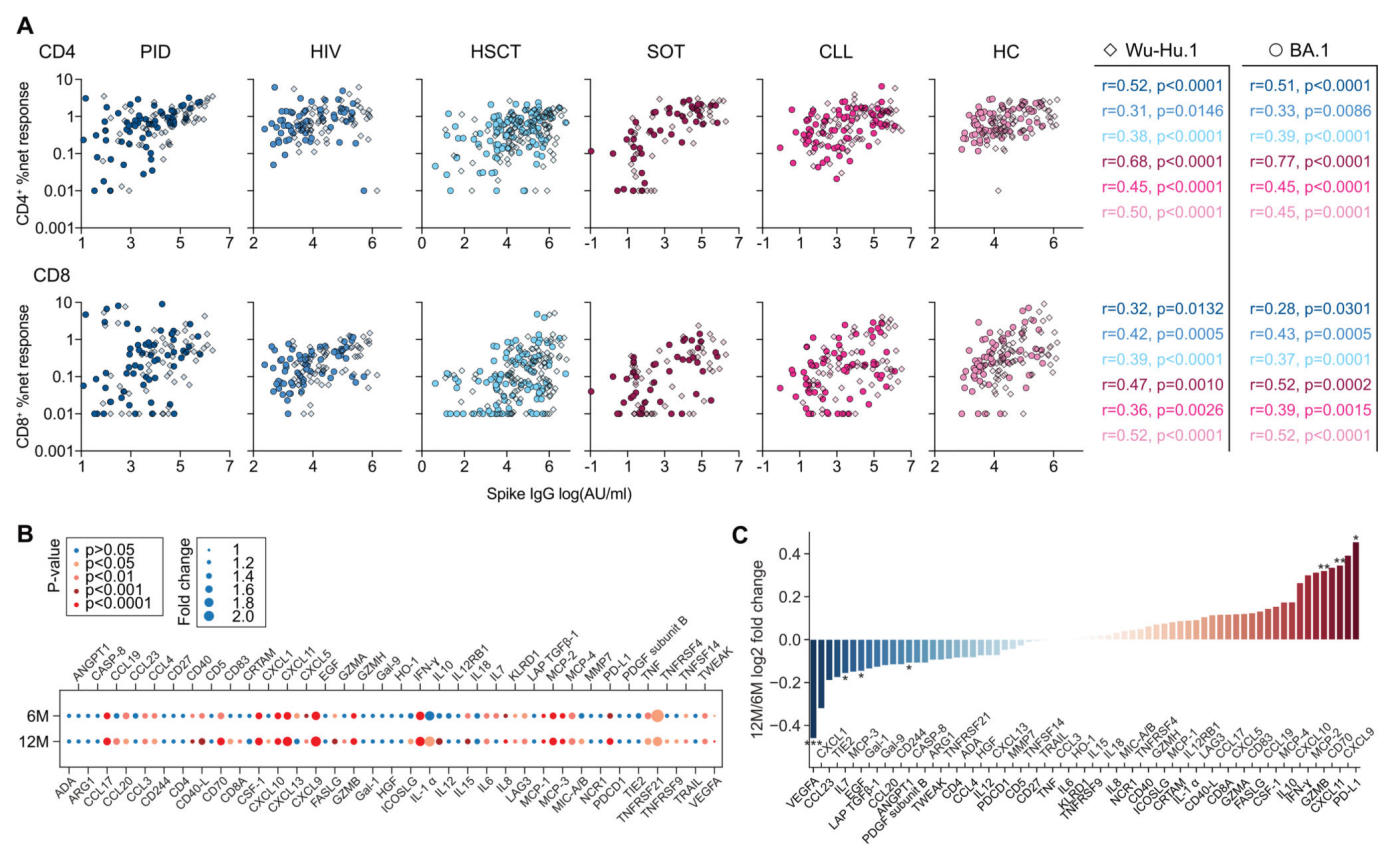
Vaccine-induced T cell responses are synchronized with antibody titers and display an inflammatory immune signature. **(A)** Correlation of anti-Wu-Hu.1 and -Omicron BA.1 spike IgG with CD4^+^ and CD8^+^ T cell responses to Wu-Hu.1 full spike and Omicron full spike peptide pools, respectively, with indicated Spearman r and p-values. Each dot represents one donor. Data are pooled over 6M and 12M time points. **(B)** Magnitude of protein secretion after Omicron full spike peptide pool stimulation of PBMCs (n=30, equally distributed across groups) before and after booster depicted as fold change over unstimulated background. **(C)** 12M/6M-ratio of data shown in (B) illustrating pre- and post-booster changes in protein secretion. (B, C) Wilcoxon matched-pairs signed rank test. **p <0.01, *p <0.05.

Next, we determined vaccination-induced anti-Wu-Hu.1 and BA.1 spike IgG titers before and after booster doses. Serological data correlated directly with T cell responses in all patient groups (Fig. 3A), particularly before booster vaccination and with CD4^+^ T cell responses (Suppl. Fig. 3A). To investigate the overall functional profile of vaccine-induced T cell responses, we measured the concentration of 92 proteins in the supernatant of Omicron full spike stimulated cells across all patient groups via proximity extension assay (26). Overall, we detected 69 proteins where about 50% were significantly different compared to negative controls (Fig. 3B). Notably increased proteins at both time points included IFN-γ, TNF, granzyme B, MCP-1/2/3/4, CD40L, CD70, CSF-1, and the CXCL9/10/11 family, which indicates a highly activated, IFN-mediated inflammatory response profile. After booster vaccination, this profile was more pronounced, as characterized by significantly increased levels of granzyme B and CXCL11, as well as a tendency of increased IFN-γ, CXCL9/10, CD70, and MCP-2/4 (Fig. 3C, Suppl. Fig. 3B), likely reflecting the observed shift in the SARS-CoV-2-specific CD8/CD4 ratio (Fig. 1D, Suppl. Fig. 1B).

### T_EMRA_ subsets with stem-like signatures are enriched in ancestral mRNA vaccine-induced CD8^+^ T cells

In addition to SARS-CoV-2-specific CD8/CD4 ratios, the composition of CD4^+^ T helper subsets, as well as CD4^+^ and CD8^+^ T cell memory phenotypes (Suppl. Fig. 4A, B for gating strategies), can give hints to the functional capacity and longevity of the vaccine-induced T cell response. In comparison to the bulk population, CD4^+^ T cells across all patient groups displayed an increased frequency of CCR6^+^CXCR3^-^ (Th17-like) and CCR6^+^CXCR3^+^ (Th1Th17-like) phenotypes and decreased frequency of CCR6^-^CXCR3^-^CCR4^+^ (Th2-like) and CXCR5^+^ (Tfh-like) subsets (Suppl. Fig. 4C). In terms of memory phenotypes, T_CM_ cells were predominant in the Omicron-reactive CD4^+^ T cell population, albeit not at different levels than the bulk population (Suppl. Fig. 4D). In the Omicron-reactive CD8^+^ T cell compartment, we observed a tendency of increased T_SCM_ and T_CM_ frequencies compared to bulk cells in all patient groups. The majority of Omicron-reactive CD8^+^ T cells showed a T_EMRA_ phenotype, but to a lower extent than the bulk population (Suppl. Fig. 4E). Investigating the effect of the booster doses on T cell subsets and memory phenotypes, we observed a trend of slightly decreasing Th1-like and increasing Th17-like subset frequencies across all patient groups (Suppl. Fig. 4F). Memory phenotypes of both CD4^+^ (Suppl. Fig. 4G) and CD8^+^ (Suppl. Fig. 4H) T cells did not significantly change after booster vaccination.

**Fig. 4:**
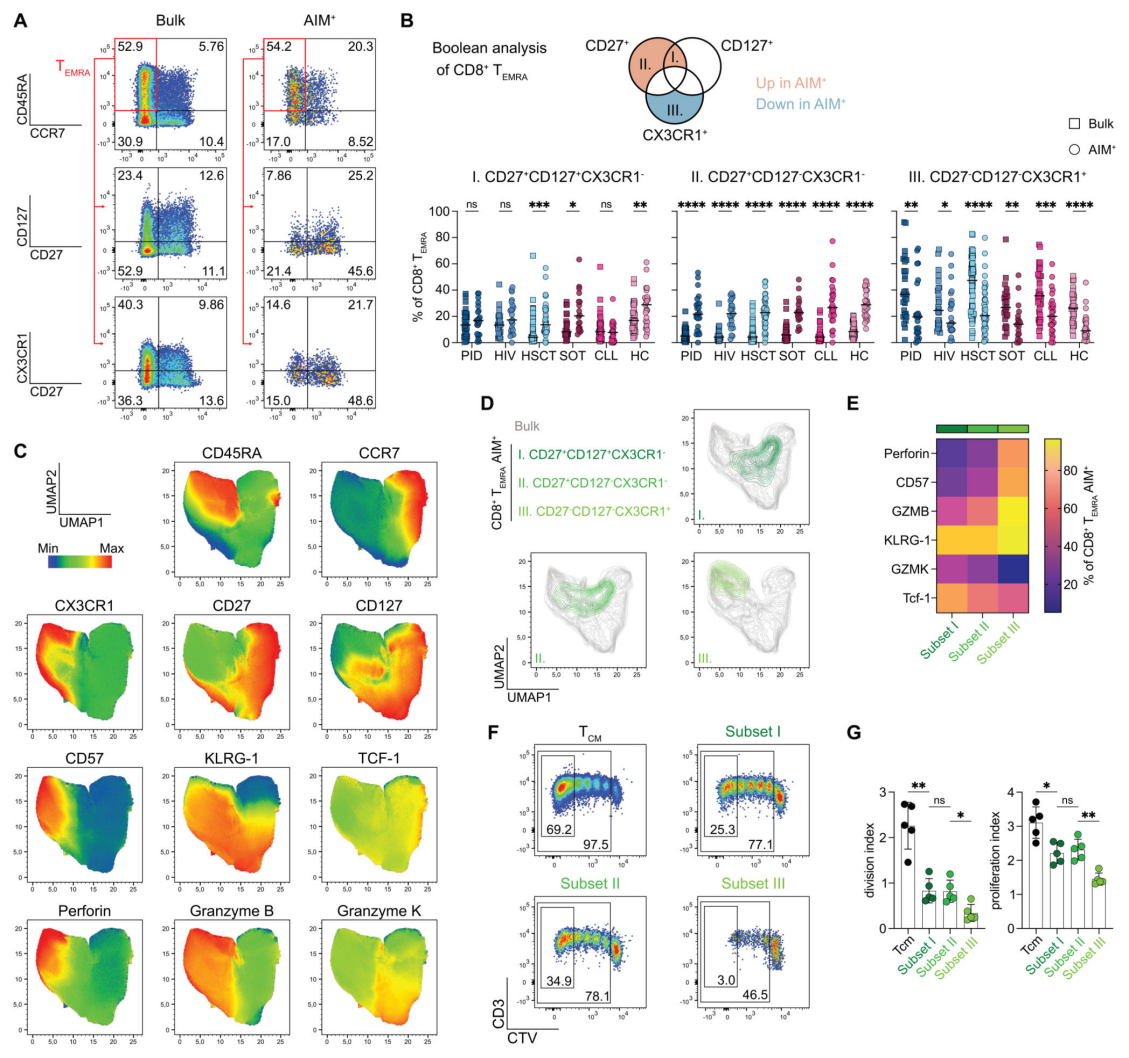
T_EMRA_ subpopulations with stem-like signatures are enriched in Omicron-reactive CD8^+^ T cells. **(A)** Representative plots show gating on CD8^+^ T_EMRA_ population as well as expression of CD27, CD127, and CX3CR1. **(B)** Boolean gating of CD8^+^ T_EMRA_ cells based on CD27, CD127, and CX3CR1 expression. Quantification shows frequencies of three different T_EMRA_ subpopulations between bulk and Omicron-reactive after booster doses. Each dot represents one donor, and lines depict the median. Wilcoxon matched-pairs signed rank test with Holm-Šidák posttest. **(C)** UMAP of bulk memory CD8^+^ T cells upon stimulation with Omicron full spike peptide pool (n=5 healthy donors, activation-markers excluded from UMAP calculation). **(D)** UMAP from (C) with an overlay of Omicron-reactive CD8^+^ T_EMRA_ subsets. **(E)** Heatmap of key differentiation markers from data shown in (C, D) (n=11 healthy donors). **(F)** Representative plots showing Cell Trace Violet (CTV) dilution after five days of CD3/CD28 stimulation of sorted CD8^+^ T_EMRA_ subsets and T_CM_ as control. **(G)** Proliferative capacity shown as division and proliferation index calculated with data measured in (F). Each dot represents one donor, and lines depict the mean ± SD. Mann-Whitney test between indicated groups. (B, G) ****p <0.0001, ^***^p <0.001, **p <0.01, *p <0.05.

We observed that about half of the vaccine-induced CD8^+^ T cell response showed a T_EMRA_ phenotype, a T cell subset characterized by its low expansion potential and limited self-renewal capacity (27). Intriguingly, however, further dissection of the CD8^+^ T_EMRA_ population revealed substantial heterogeneity based on the expression of the late differentiation marker CX3CR1 but also of early-differentiation (stem-like) markers CD27 and CD127 (Fig. 4A, Suppl. Fig. 5A). Boolean analysis highlighted three different subsets within the Omicron spike-reactive CD8^+^ T_EMRA_ compartment based on size and deviation from bulk CD8^+^ T_EMRA_ (Fig. 4B, Suppl. Fig. 5B). CD27^+^CD127^+^CX3CR1^-^ (subset I) and CD27^+^CD127^-^CX3CR1^-^ (subset II) Omicron spike-reactive CD8^+^ T_EMRA_ cells were significantly enriched, whereas a CD27^-^CD127^-^CX3CR1^+^ population (subset III) was significantly diminished compared to bulk CD8^+^ T_EMRA_ cells. Comparing frequencies between patient groups showed diminished subset I in CLL, no significant differences of subset II, and slightly increased subset III in HSCT and CLL patients (Suppl. Fig. 5C). Overall, administration of booster doses did not significantly alter CD8^+^ T_EMRA_ subset frequencies, although we observed a trend of increased subset II frequencies across patient groups (Suppl. Fig. 5D).

To further explore differences in functionality and differentiation state between these spike-reactive CD8^+^ T_EMRA_ subsets, we combined the AIM assay with intracellular staining of granzyme B, granzyme K, perforin, TCF-1, and additional surface staining of CD57 and KLRG-1 (Fig. 4C). Uniform Manifold Approximation and Projection (UMAP) with an overlay of the three spike-reactive CD8^+^ T_EMRA_ subsets on the bulk population (Fig. 4D) as well as quantification of expression levels (Fig. 4E) shows gradually increasing TCF-1 and granzyme K from subset III to subset I. Vice versa, expression levels of late differentiation markers perforin, CD57, and granzyme B gradually decrease from subset III to subset I. These data indicate that a heterogenous CD8^+^ T_EMRA_ compartment comprises a range of stem-like (subsets I and II) to terminally differentiated cells (subset III). To confirm this on a functional level, we assessed the proliferative capacity of these subsets. We labeled PBMCs with a stable fluorescent dye, flow-sorted subsets I, II, III, and CD8^+^ T_CM_ cells (as positive control), and subsequently performed polyclonal *in vitro* stimulation (Fig. 4F). Highest proliferation capacity was observed in T_CM_ cells as expected. Subsets I and II showed similar proliferation capacity that was significantly higher compared to subset III (Fig. 4G). Collectively, our data indicate substantial heterogeneity within the spike-reactive CD8^+^ T_EMRA_ population, including significantly enriched stem-like subsets with increased proliferative capacity (subsets I and II) as well as a significantly decreased subset of bona fide terminally differentiated cells (subset III) after SARS-CoV-2 mRNA vaccination.

### Infection induces Omicron spike-specific responses in booster-vaccinated individuals

**Fig. 5:**
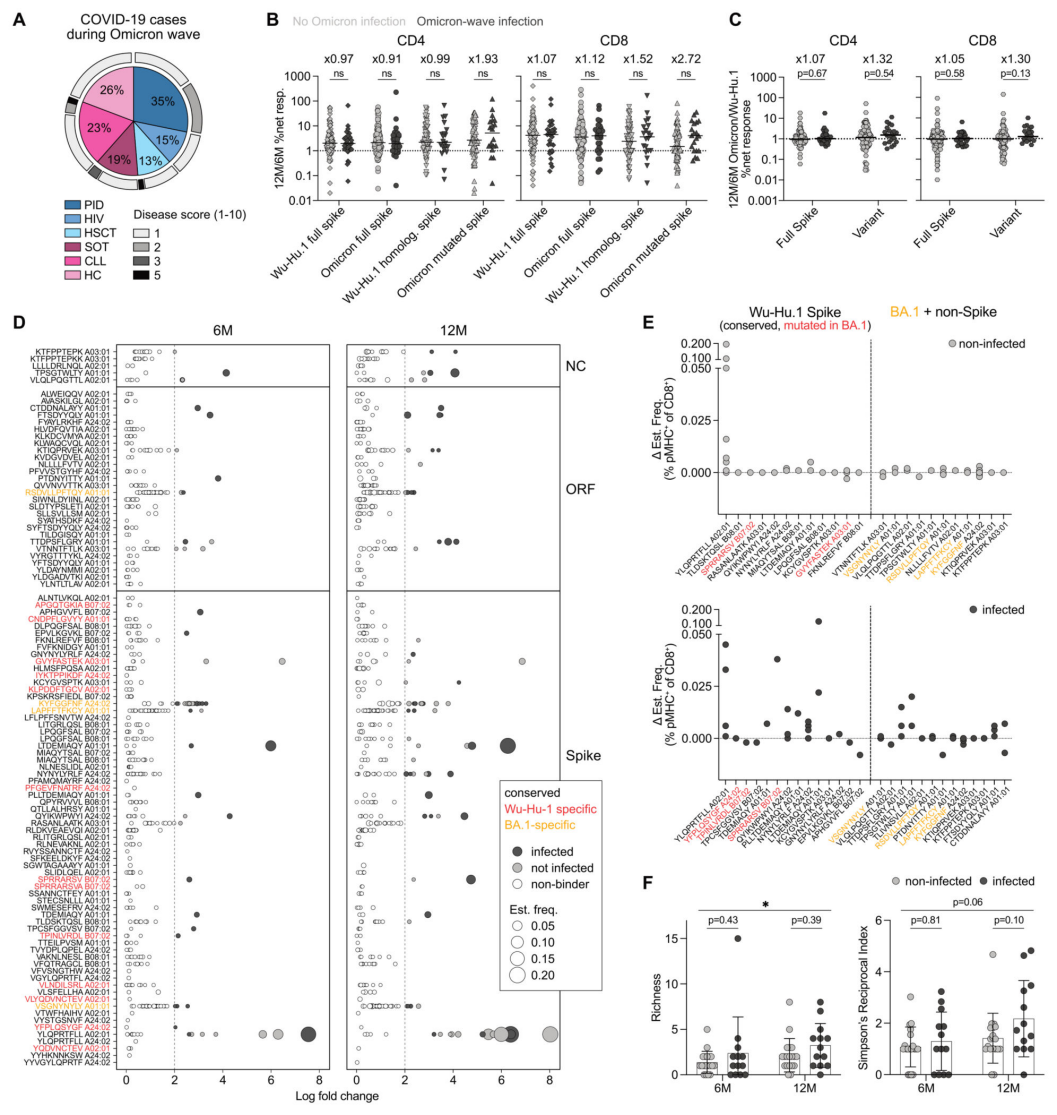
Omicron infection induces T cell responses to Omicron spike and non-spike epitopes. **(A)** Distribution of cases between patient groups and disease grade of infected individuals during the Omicron wave of the entire COVAXID cohort (n=356). Disease score according to WHO clinical progression scale (32). Percentages of cases in each individual patient group are indicated. **(B)** Ratio of 12M/6M T cell responses to Wu-Hu.1 and Omicron spike peptide pools in Omicron-wave infected and non-infected individuals. **(C)** Full spike and variant peptide pool Omicron/Wu-Hu.1-ratios of 12M/6M T cell responses. **(D)** Binding of pMHC-multimers across nucleocapsid (NC), open reading frame (ORF), and spike epitopes restricted to HLA-A01:01, -A02:01, -A03:01, A24:02, -B07:02, and -B08:01 depicted as log fold change over background (n=23 non-infected and n=13 infected, evenly distributed across groups except HIV) **(E)** Magnitude difference of detected pMHC-binder frequencies between 12M and 6M time points for Wu-Hu.1 spike versus Omicron BA.1 and non-spike epitopes. **(F)** Calculated Richness and Simpson’s Reciprocal Index of all detected pMHC-binders (spike and non-spike, conserved, Wu-Hu.1-specific and Omicron BA.1-specific). Lines depict mean ± SD. Student’s t-test for comparison of time points with pooled data. Two-way ANOVA with Šidák posttest for comparison of non-infected versus infected. *p <0.05. (B, C) Data are pooled across all patient groups. Numbers indicate median fold changes. Mann-Whitney test with Holm-Šidák posttest. (B, C, F) One dot represents one patient. (D to F) Data are pooled across all patient groups except HIV. (D, E) Each dot represents a binding response to one individual pMHC-multimer.

Study participants received their third dose before the onset of the Omicron BA.1 variant in Sweden. This opened the opportunity to assess vaccine-induced protection from severe Omicron disease across various immunodeficiencies and investigate T cell responses to this variant. Of the entire COVAXID cohort, 78/356 (22%) individuals were infected during the Omicron wave, as confirmed by polymerase chain reaction or rapid antigen test. The number of infections was comparative between patient groups and ranged between 13% and 35%. Notably, only two patients developed moderate disease and required hospitalization, whereas all others experienced only mild symptoms (Fig. 5A). Blood samples for analysis of cellular responses were available from 38 infected individuals similarly distributed across patient groups. Comparing clinical and demographic data of infected and uninfected individuals, no notable differences in clinical or demographic data were observable (Suppl. Table 2). To assess T cell responses against Wu-Hu.1 and Omicron spike, we performed the AIM assay with peptide pools covering the entire spike protein of Wu-Hu.1 and Omicron but also with smaller (i.e., comprising comparably fewer peptides) variant peptide pools only containing Omicron-mutated spike peptides and their homologous Wu-Hu.1 counterparts. T cell responses reactive to the full spike and Wu-Hu.1 homologous variant pool increased similarly in infected and non-infected individuals between the 6-month and 12-month time points. However, the median CD4^+^ and CD8^+^ T cell responses to the Omicron mutated spike peptide pool were 1.93- and 2.72-times higher in infected individuals, respectively (Fig. 5B, see Suppl. Fig. 6A for individual patient group data). We observed increased Omicron-mutated spike-specific T cell responses in Omicron-wave infected individuals across all patient groups (Suppl. Fig. 6B). Comparing Omicron spike-specific responses between groups, HSCT patients appeared to mount lower T cell responses, but these results should be interpreted cautiously due to low sample numbers (Suppl. Fig. 6C). Comparing relative response increases over time between Wu-Hu.1 and Omicron peptide pools, a minor increase in infected individuals was observed for the full spike pools. This increase was more pronounced in the smaller variant pools, indicating that Omicron infection generates T cell responses that specifically recognize Omicron spike epitopes (Fig. 5C).

### Booster-vaccinated individuals develop a more diverse SARS-CoV-2-reactive T cell repertoire upon Omicron infection

Next, we wanted to investigate Omicron infection-induced T cell repertoire perturbations on the epitope level. We performed epitope mapping as previously described (28), using a large set of barcoded peptide-major histocompatibility complex (pMHC)-multimers for immunogenic Wu-Hu.1 spike, open reading frame (ORF), nucleocapsid (NC), and Omicron-specific (spike, ORF, NC) epitopes restricted to HLA-A01:01, -A02:01, -A03:01, -A24:02, - B07:02, and -B08:01 (representing the most common HLA-types in the European-Caucasian population). pMHC-multimer pools contained previously identified immunogenic Wu-Hu.1 epitopes (28, 29), including n=82 spike, n=5 NC, and n=32 ORF pMHCs. Further, we used NetMHCpan 4.1 (30) to predict Omicron spike epitopes and selected n=38 to generate barcoded pMHCs (a list of all pMHCs is provided in Suppl. Tables 3-5). Samples from infected and non-infected (n=13 and 23, respectively, evenly distributed across groups except HIV) were stained with pMHC-multimer pools. Subsequently, pMHC-positive cells were sorted on a flow cytometer and sequenced for pMHC-barcode identification. Epitope-specific T cell recognition is identified as ≥ two log-fold change enrichment of pMHC-barcodes than the background (and p<0.001), and the total barcode reads associated with a significant pMHC binding was used to calculate estimated T cell frequencies in each sample. We detected a substantial number of pMHC-binders and confirmed our previously published results (31) on highly immunogenic SARS-CoV-2 Wu-Hu.1 epitopes, such as the prevalent YLQPRTFLL (S269-277) HLA-A02:01 response (Fig. 5D). Overall, the majority of detected binders corresponded to conserved epitopes, with only a few epitopes specific for either Wu-Hu.1 or Omicron BA.1. Additionally, we observed 45% more pMHC-binders at 12 months compared to 6 months, where the average frequency was larger by a factor of 1.8, aligning with the observed increases using the AIM assay. Analysis of 6-month to 12-month frequency changes of pMHC-binders to either Wu-Hu.1 spike or Omicron BA.1 and non-spike revealed distinctly more changes in infected individuals, particularly in response to conserved spike and non-spike epitopes (Fig. 5E). Increased frequencies of non-spike pMHC-binders in infected individuals confirmed infection history. Importantly, both booster vaccination and additional Omicron infection increased the diversity of the SARS-CoV-2-reactive T cell repertoire, as shown by increased Richness and Simpson diversity (Fig. 5F). Collectively, Omicron-infected individuals displayed increased T cell responses to Omicron-mutated spike (Fig. 5B, C) and a more diverse SARS-CoV-2-reactive T cell repertoire through a more even response to spike, and generation of new T cell responses to targets that were absent in ancestral-based mRNA spike vaccines.

## Discussion

The presence of SARS-CoV-2-reactive T cells limits viral load in animal models (6) and likely confers protection from severe disease in humans (33). Moreover, the magnitude of SARS-CoV-2 Wu-Hu.1 vaccine-elicited T cells correlates with decreased risk of symptomatic COVID-19 in humans, extending to the Delta and Omicron variants (7). Most healthy immunocompetent individuals generate potent SARS-CoV-2-specific T cell responses after two vaccine doses (34, 35) comparable to levels observed upon SARS-CoV-2 infection (8). However, individuals across different immunodeficient states display impaired vaccine effectiveness (17) and frequently generate low to non-detectable T cell responses upon two SARS-CoV-2 mRNA vaccine doses (18). Consequently, immunocompromised patients were shown to have increased COVID-19-related mortality and are considered to be a high-risk group (20). Here, we show that SARS-CoV-2 spike-reactive T cell responses upon two vaccine doses are variable between different immunodeficient patient groups and particularly low in transplant recipients and CLL patients receiving immunosuppressive medication, confirming earlier studies reporting impaired vaccine-induced disease protection in this patient group (17, 19). Importantly, we detected markedly increased CD4^+^ and CD8^+^ T cell responses upon additional booster vaccination as observed previously (9, 36) but also contrasting another recent report where T cell responses remained stable (37). Whereas pre-booster T cell responses showed high inter-individual and inter-patient group variability, post-booster responses reached more similar levels. More detailed analyses revealed that poor responders after two doses particularly benefit from booster vaccination and that CD8^+^ T cell responses, in many cases being not detectable before the booster, increase distinctly more than CD4^+^ T cells. These observations suggest the presence of a ceiling effect, in which both antibody and T cell response magnitudes saturate, and is in line with observations in previous reports (24, 38, 39). Moreover, poor vaccination responsiveness due to age and immunodeficiency could be counteracted through the administration of additional vaccine doses. Hence, our data clearly underline the added benefit of booster vaccination for building up robust spike-specific T cell responses, especially in immunocompromised individuals and those who responded poorly to the first two doses.

Repetitive stimulation of the same T cell population through vaccination might negatively affect T cell memory phenotypes and, thereby, longevity and functional capacity (40). Similar to what has been observed in convalescent, as well as vaccinated infection-naïve healthy individuals (41, 42), we observed a T_CM_-dominated CD4^+^ spike-specific T cell population and a tendency of increased T_SCM/CM_ T cell frequencies in the CD8^+^ compartment. The increased presence of stem-like CD8^+^ T cells has been linked to high polyfunctionality, proliferative capacity, and persistence (43). Earlier observations of increased memory transcripts (such as IL7R, MYC, and BCL2L1) in single-cell sequencing data of spike-specific CD8^+^ T_EMRA_ cells after full SARS-CoV-2 mRNA vaccination (18) prompted us to investigate this population more thoroughly. Performing the AIM assay along with a large staining panel of memory and differentiation surface markers, we observed substantial heterogeneity in the spike-specific CD8^+^ T_EMRA_ population. Stem-like CD27^+^ and CD127^+^ CD8^+^ T_EMRA_ subsets were substantially increased compared to bulk CD8^+^ T_EMRA_ across patient groups, whereas terminally differentiated CX3CR1^+^ cells were markedly decreased, both before and after booster vaccination. These findings align with our previous data, and similar observations have also been reported after natural infection (44). Further investigations showed that CD27^-^CD127^-^ CX3CR1^+^ cells also expressed the late terminal differentiation marker CD57, displayed the highest expression of perforin and granzyme B, and lowest proliferative capacity. In contrast, CD27^+^CD127^+/-^CX3CR1^-^ cells expressed higher levels of TCF-1 and granzyme K and proliferated distinctly more upon stimulation, indicating stem-like characteristics. Hence, a substantial portion of vaccination-induced spike-specific CD8^+^ T_EMRA_ cells display retained potency giving little indication of dysfunctionality through repetitive stimulation.

Another potential adverse effect of repetitive administrations of the ancestral strain-based vaccine could be the stimulation and expansion of Wu-Hu.1 spike-specific T cell clones that do not sufficiently respond to Omicron-mutated epitopes but block the formation of new Omicron-specific T cell clones. We and others have shown that T cell responses to the spike peptidome of Omicron and other VOCs are maintained in healthy convalescent and fully vaccinated individuals (8–10, 45), in contrast to diminished neutralizing antibody responses to Omicron (1, 2). However, a recent study with a smaller cohort reported completely abrogated T cell responses to Omicron in healthy triple-vaccinated, infection-naïve individuals (46). This study also reported significantly decreased Omicron reactivity in triple-vaccinated individuals who experienced earlier infections with the ancestral Wu-Hu.1 strain followed by Omicron B.1.1.529 and proposed a relation to the antigenic sin effect (12, 13). In this study, measurement of Wu-Hu.1 versus Omicron total spike-reactive T cells via AIM assay showed that Omicron T cell responses are significantly increased after booster vaccination and comparable to Wu-Hu.1 responses. Accordingly, and quite encouragingly, within our large cohort and across all immunodeficiency states, 78 individuals (22%) were infected during the Omicron wave, but no individuals developed severe disease, and most patients experienced mild symptoms despite various underlying immunodeficiencies. These data are in line with a recently published meta-study that reports additive effects of booster vaccination and hybrid immunity on Omicron re-infection risk and protection from severe disease (>95%) (5). Whereas potent T cell responses have been shown to confer protection from severe COVID-19 (6, 7, 33), there is also evidence that Omicron variants are less pathogenic (47, 48). As such, it is highly likely that both increased pre-existing SARS-CoV-2 immunity and decreased virus pathogenicity contribute to maintaining the number of severe cases lower than those observed with previous variants (49).

To decipher vaccine-induced and Omicron infection-induced T cell responses, we performed the AIM assay using the smaller Omicron-mutated spike peptide pool and its Wu-Hu.1 counterpart, which enabled us to examine the induction of new Omicron-specific T cell responses. Importantly, increased responses to Omicron-specific spike epitopes were observed in previously Omicron-infected individuals, as observed in other cohorts of immunocompromised patients (50, 51). However, the differences were small, indicating a solid immunological imprint derived from three to four Wu-Hu.1-based vaccine doses. Nevertheless, even small numbers of Omicron-specific T cells might be boosted and expanded to higher frequencies upon a subsequent infection, further strengthening and diversifying the overall SARS-CoV-2-reactive T cell repertoire. This is in line with recent data showing that individuals with hybrid immunity from vaccination and previous infection had the highest protection from symptomatic Omicron infection, particularly when the previous infection was also caused by Omicron (52). To investigate the T cell repertoire more closely, we generated a selected number of HLA-A01:01, -A02:01, -A03:01, -A24:02, -B07:02, and -B08:01 Wu-Hu.1 spike-, NC-, ORF-, and Omicron spike-specific multimers. DNA-barcoding of these multimers allowed pooling for an intermediate-throughput screening approach. We only observed a limited number of Omicron-specific pMHC-binders, which might be explained by a relatively low number of applied pMHC-multimers and potentially low frequencies of infection-induced Omicron-specific responses. More strikingly, however, booster-vaccinated and Omicron-wave-infected individuals showed a more diverse response pattern to conserved non-spike and spike epitopes. These data confirm their infection history and show that Omicron infection induces new T cell clones and diversifies the overall SARS-CoV-2-reactive T cell repertoire. These findings are based on a relatively low number of Omicron-infected individuals within our otherwise large cohort, representing a limitation of this study. Epitope mapping is complicated by the extremely high diversity of epitope-HLA combinations. Larger samples numbers would help to overcome this restriction and generate higher resolving data of TCR repertoire perturbations upon Omicron infection.

In summary, we demonstrate the additive effects of ancestral-based booster vaccination and Omicron infection on cellular immunity across different immunocompromised states. Our findings provide evidence that additional booster vaccine doses secure strong induction of potent Omicron-reactive CD4^+^ and CD8^+^ T cell responses, even in immunocompromised individuals with initial poor vaccination responsiveness. T cell responses displayed an inflammatory signature, signs of longevity, and were synchronized with antibody titers. Upon repetitive mRNA vaccine doses and Omicron infection, we observed enhanced and diversified T cell responses. As such, these data suggest minimal indications of antigenic sin effects or functional exhaustion of T cell immunity despite repetitive antigen exposure and solid immunological imprints by ancestral SARS-CoV-2 mRNA vaccination.

## Materials and Methods

### Study design

The objective of this study was to characterize the SARS-CoV-2-specific T cells response following mRNA COVID-19 booster vaccination as well as additional Omicron infection in healthy and immunocompromised individuals.

### Human subjects, sample collection, and ethics

In this prospective longitudinal clinical trial study, healthy controls and patients with primary immunodeficiency (PID), human immunodeficiency virus infection type 1 (HIV), hematopoietic stem cell transplantation (HSCT), solid organ transplantation (SOT), and chronic lymphocytic leukemia (CLL) were recruited between February to March 2021. Detailed patient characteristics have been described elsewhere for the entire COVAXID study (23). The original clinical trial protocol included two vaccine doses and immunogenicity measurements until six months after the first dose and was subsequently extended with permission from the Swedish Ethical Review Board and the Swedish Medical Products Agency (no. 2021-06046-02 and no. 5.1-2021-92151, respectively). 356 of 539 (66.0%) study participants consented to continued participation in the extended clinical trial. From about ½ of the study participants, additional blood samples for analysis of cellular responses were collected immediately before the first vaccine dose (Day 0), six months (6M), and twelve months (12M). Booster vaccination occurred between the six-month and twelve-month time points. The third and, in applicable cases, fourth mRNA vaccine dose was scheduled following recommendations by the Public Health Agency of Sweden. In this context, patient groups considered the most severely immunocompromised were offered a third vaccine dose, in most cases between months 6 and 9, and a subsequent fourth dose between months 9 and 12. In most cases, other patients and controls were offered the third dose between months 9 and 12. Verified SARS-CoV-2 infection was recorded in an electronic case report form (eCRF). The trial was registered at EudraCT (no. 2021-000175-37) and clinicaltrials.gov (NCT04780659). The Swedish Medical Product Agency (ID 5.1-2021-5881) and the Swedish Ethical Review Authority (ID 2021-00451) approved the study. PBMCs were isolated from whole blood via standard density gradient centrifugation and cryopreserved in fetal bovine serum (FBS) containing 10% dimethyl sulfoxide (DMSO). All participants have given their written informed consent to the study.

### Peptides

Peptide pool stimulations used 316 and 315 overlapping peptides (15-mers with 11 aa overlap) (Peptides&Elephants) from the entire SARS-CoV-2 spike glycoprotein (UniProt: P0DTC2) derived from the ancestral Wuhan strain or Omicron B.1.1.529 respectively. To assess Omicron-specific T cell responses, peptide pool stimulations used 80 and 82 peptides (15-mers) (Peptides&Elephants) from Omicron B.1.1.529-mutated regions within SARS-CoV-2 spike glycoprotein and Wu-Hu.1 homologous counterparts. The spike peptide pools were reconstituted in DMSO, diluted to 100 μg/ml in PBS, aliquoted, and stored at −20°C.

### Activation-Induced Marker (AIM) assay

Cryopreserved PBMCs were thawed quickly, resuspended in complete medium (RPMI 1640 containing 10% FBS, 1% L-glutamine, and 1% penicillin/streptomycin) supplemented with DNase I (10 U/ml; Sigma-Aldrich), and rested at 1×10^6^ to 2x10^6^ cells per well in 96-well U-bottom plates (Corning) for 3 hours at 37°C. The medium was then supplemented with anti-CXCR5 (clone RF8B2; BD Biosciences) and anti-CD40 (unconjugated, clone HB14, Miltenyi) followed 15 min later by the respective spike peptide pool (0.5 μg/ml) for stimulation or an equivalent amount of DMSO as a negative control. After 12 hours, cells were washed in PBS supplemented with 2% FBS and 2 mM EDTA (FACS buffer) and stained with other chemokine receptors (CCR6, CCR4, CCR7, CXCR3, CX3CR1) for 10 min at 37°C. Additional staining of surface molecules (CD8, CD45RA, CD14, CD19, CD127, CD3, CD27, CD95, CD4), including activation markers CD69, CD40L, and 4-1BB, was performed for 30 min at room temperature in the presence of Brilliant Stain Buffer Plus (BD Biosciences). Viable cells were identified by exclusion using a LIVE/DEAD Fixable Aqua Dead Cell Stain Kit (Thermo Fisher Scientific). Stained cells were washed in FACS buffer, fixed in PBS containing 1% paraformaldehyde (PFA; Biotium), and acquired using a FACSymphony A5 (BD Biosciences). All antibodies are listed in Table S6.

Net frequencies of spike-reactive T cells were calculated by subtracting the frequency of AIM marker^+^ T cells in the negative control from the frequency of AIM marker^+^ T cells detected after stimulation with each peptide pool, with values smaller than 0.01% set to 0.01%. Stimulation indices were calculated as fold change. Positive responses were defined based on the requirement of a stimulation index ≥2 and a minimum of ten cells in the AIM marker^+^ gate as described previously (53).

### Antibody tests

Plasma samples were tested for IgG binding to SARS-CoV-2 spike Wu-Hu.1 and Omicron variants B1.1.529/BA.1 using V-PLEX SARS-CoV-2 panels 2 and 25 (Meso Scale Diagnostics, MSD). The assays were performed according to the manufacturer’s instructions at the SciLifeLab Affinity Proteomics Unit in Uppsala, Sweden. Antibody titers were expressed as arbitrary units (AU)/ml.

### AIM assay with intracellular staining

Stimulation and staining procedure as described for the AIM assay. Chemokine receptors (CCR7, CX3CR1) were stained for 10 min at 37°C followed by staining of surface markers (CD8, CD45RA, CD14, CD19, CD127, KLRG-1, CD69, CD3, CD57, CD95, CD27, 4-1BB, CD4) for 30 min at room temperature in the presence of Brilliant Stain Buffer Plus (BD Biosciences). Cells were then washed in FACS buffer and fixed/permeabilized using a FoxP3 Transcription Factor Staining Buffer Set (Thermo Fisher Scientific). Intracellular stains (granzyme B, perforin, TCF-1, granzyme K) were performed for 30 min at room temperature. Stained cells were washed in FACS buffer and acquired using a FACSymphony A5 (BD Biosciences). All antibodies are listed in Table S6.

### Serum protein quantification using proximity extension assay

Culture supernatants from Omicron full spike peptide pool and negative control stimulations from the AIM assay were harvested immediately after the 12-hour stimulation and stored at -80°C until sent for analysis. 92 proteins from the Immuno-Oncology Olink panel were assessed using proximity extension assay technology (Olink AB) on paired samples from six-month and twelve-month time points of six individuals from each patient group.

Data were analyzed using Python (v.3.8.8), pandas (v.1.2.4), NumPy (v.1.20.1), and Scipy (v.1.8.1). For the analysis of stimulated vs. unstimulated conditions, mean fold changes were calculated from raw data for each analyte and values were compared using matched Wilcoxon test. For the analysis of changes between 12 and 6 months, Olink values were background subtracted and normalized using the MinMaxScaler function from scikit-learn (v.1.1.1). Normalized values were used for calculating mean fold changes, and values were compared using matched Wilcoxon test. Visualization was done using seaborn (v.0.11.1) and matplotlib (v.3.3.4).

### Cell sorting and proliferation assay

Cryopreserved PBMCs were thawed quickly, resuspended in complete medium in the presence of DNase I (10 U/ml; Sigma-Aldrich), and rested overnight at 1×10^6^ cells/ml complete medium supplemented with 20 IU/ml IL-2. Cells were washed with pre-warmed (37°C) PBS and subsequently incubated in a 2.5 μM Cell Trace Violet (Thermo Fisher Scientific) solution for 10 minutes at 37°C. After incubation, cells were repeatedly washed with complete medium. Following the CTV labeling, CD8 T cells were enriched via immunomagnetic negative selection using the EasySep™ Human CD8^+^ Cell Enrichment Kit (STEMCELL Technologies). Enriched cells were counted and stained for chemokine receptors (CCR7, CX3CR1) and surface markers (CD8, CD45RA, CD14, CD19, CD127, CD3, CD27, CD95, CD4) as described for the AIM assay. Stained cells were then washed, resuspended in FACS buffer, and sorted using a FACSAria™ (BD Biosciences). Sorted CD8 T_CM_, T_EMRA_ CD27^+^CD127^+^CX3CR1^-^, T_EMRA_ CD27^+^CD127^-^ CX3CR1^-^, and T_EMRA_ CD27^-^CD127^-^CX3CR1^+^ were transferred to a 96-well U-bottom plate (Corning) and stimulated with 5μl/ml CD3/CD28/CD2 mix (Immunocult, STEMCELL Technologies) in complete medium supplemented with 20 IU/ml IL-2 for five days. Subsequently, cells were stained for chemokine receptors (CCR7, CX3CR1) and surface markers (CD8, CD45RA, CD14, CD19, CD127, KLRG-1, CD69, CD3, CD27, CD95, 4-1BB, CD4) according to procedure described for the AIM assay. All antibodies are listed in Table S6.

### MHC-I monomer production

The six different MHC class I molecules were produced as described previously (54, 55). In short, MHC-I heavy chain for these HLA types and human β2-microglobulin (hβ2m) light chain were expressed in *Escherichia coli* BL21(DE3)pLysS (Novagen 69451) using pET series expression plasmids, and soluble denatured proteins were harvested from inclusion bodies. Soluble proteins were folded and purified *in vitro* either using UV-sensitive HLA-specific peptide ligands (HLA-A01:01, -03:01, -B07:02, and -B08:01) (56) or empty (HLA-A02:01, and -24:02) (57). Monomers were subsequently biotinylated using a reaction kit (Avidity LLC) and purified by size exclusion chromatography (SE-HPLC).

### SARS-CoV-2 peptide selection

For antigen-specific CD8 T-cell analysis a total of 160 SARS-CoV-2 derived peptides restricted to six MHC class I allotypes (HLA-A01:01, -A02:01, -A03:01, -A24:02, -B07:02, and -B08:01) were selected. 122 of these peptides were shown to be immunogenic in SARS-CoV-2 infection (n=56) (wild type variant) or vaccination (n=66, SARS-CoV-2 spike protein) in our previous analysis (28, 29). Peptides (n=38) within the Omicron variant (B.1.1.529) of SARS-CoV-2 were selected based on mutations observed between the Omicron variant and the ancestral Wuhan strain and predicted to bind one or more of the six MHC class I allotypes.

The peptides were synthesized by Pepscan (Pepscan Presto BV, Lelystad, Netherlands) at 2 µmol scale and quality controlled by mass spectrometry analysis. All the peptides were dissolved to 10 mM in dimethyl sulfoxide (DMSO; Sigma Aldrich D2650) and stored at -20°C until use.

### DNA-barcoded pMHC-multimer library preparation

SARS-CoV-2 pMHC libraries were generated by incubating 200 µM of each peptide with 100 µg/ml of the respective MHC molecules for 1 hour using UV-mediated peptide exchange (HLA-A01:01, A03:01, B07:02, B08:01) or direct binding to empty MHC molecules (HLA-A02:01 and A24:02). HLA-specific DNA-barcoded multimer libraries were then generated by incubating pMHC monomers to their corresponding DNA barcode-labeled dextrans at 4°C for 30 min. The DNA barcode-labeled dextrans were prepared as previously described (56). In short, barcodes, resembling a 5’ biotinylated unique DNA sequence, were attached to a streptavidin-conjugated dextran backbones (Fina Biosolutions, Rockville, MD, USA), which were either fused to phycoerythrin (PE) or allophycocyanin (APC), by incubating them at 4°C for 30 min to generate a unique DNA barcode dextran library.

### pMHC-multimer staining and barcode sequencing

PBMCs were thawed in RPMI (Gibco 72400021) media supplemented with 10% fetal bovine serum (FCS; Gibco, 10500064), 100 µg/ml DNAse I (StemCell Technologies 07470), and 5mM MgCl_2_. Cells were then washed twice in RPMI + 10% FBS and once more in barcode cytometry buffer (BCB; PBS + 0.5% BSA + 100 μg/mL herring DNA + 2 mM EDTA). pMHC-multimer carrying SARS-CoV-2 wildtype and Omicron peptides were pooled and incubated with 5-10x10^6^ PBMCs for 15 min at 37°C followed by incubation at 4 °C for 30 min with a phenotype antibody panel containing surface marker antibodies (CD3, CD4, CD8) and a dead cell marker (LIVE/DEAD Fixable Near-IR; Invitrogen, L10119) (final dilution 1/1000). Cells were washed twice with barcode cytometry buffer and fixed in 1% paraformaldehyde.

Cells were then acquired on a FACSAria flow cytometer (AriaFusion, Becton Dickinson). All SARS-CoV-2 Wu-Hu.1 multimer binding and Omicron B.1.1.529 variant multimer binding CD8^+^ T cells were sorted into pre-saturated tubes (2% bovine serum albumin and 100 µl of barcode cytometry buffer). Subsequently, sorted cells were subjected to polymerase chain reaction amplification of associated DNA barcodes. DNA barcodes from the samples and DNA barcodes from an aliquot of the initial multimer pool (10,000x final dilution in the PCR reaction, which was used as a baseline) were PCR-amplified using the Taq PCR Master Mix Kit (Qiagen, 201443). PCR products were purified with the use of QIAquick PCR Purification kit (Qiagen 28104) and sequenced at PrimBio (USA) using an Ion Torrent PGM 318, or an Ion S5 530 chip (Life Technologies).

### DNA barcode sequence analysis and identification of pMHC specificities

The sequencing data were processed using the Barracoda software package (https://services.healthtech.dtu.dk/service.php?Barracoda-1.8). This enables calculating the number of reads and clonally reduced reads for each DNA barcode that is associated with a specific pMHC, the fold change (FC) in read counts mapped to a given sample relative to the mean read counts mapped to triplicate baseline samples, the p-values, and the false-discovery rates (FDRs) (56). DNA barcodes with p < 0.001 and Log2 fold change >2 over the baseline values for the total pMHC library were considered significant and defined as a pMHC binder. Each significantly enriched barcode, representing T cell frequency, was calculated based on the percentage of CD8^+^pMHC^+^ T cells and the percentage of read count of the barcode within that population. Peptides recognized in a non-HLA-matching healthy donor, which was included as a negative control, were subtracted from the data set to exclude non-specific pMHC binding to T cells.

### Data analysis

Flow cytometry data were analyzed using FlowJo software version 10.7.1 (FlowJo LLC). Dimensionality reduction was performed using the FlowJo plugin UMAP version 3.1 (FlowJo LLC). Down-sampled files concatenated from representative donors (n=5) were used for these analyses with default settings (distance function: Euclidean; nearest neighbors: 15; minimum distance: 0.5) for the indicated markers (Fig. 4, Suppl. Fig. 5). Output from the DNA-barcoded pMHC-multimers analysis and Barracoda software, was plotted using RStudio version 4.1.0. Statistical analyses were performed using Prism version 9 (GraphPad Software Inc.) using significance tests as indicated in respective figure legends.

## Supplementary Material

Supplementary Figures and Tables

## Data Availability

All data needed to evaluate the conclusions of this publication are present in the paper or the Supplementary Materials. Any additional information required to reanalyze the data reported in this paper is available from the corresponding authors upon reasonable request.

## One sentence summary

Omicron-reactive T cell responses expand upon booster vaccination and diversify upon Omicron infection
