# Supplementary material for: Additive effects of booster mRNA vaccination and SARS-CoV-2 Omicron infection on T cell immunity across immunocompromised states
Source: Sci Transl Med. Author manuscript; Available in PMC 2024 Feb 8. (PMC7615622; doi:10.1126/scitranslmed.adg9452)
Supplement: Supplementary Figures and Tables [file EMS193806-supplement-Supplementary_Figures_and_Tables.pdf]

LIST OF SUPPLEMENTARY MATERIALS

Suppl. Fig. 1. T cell responses to ancestral mRNA vaccine doses over time.

Suppl. Fig. 2. Individual variability of vaccine-induced Omicron-reactive T cell responses based on disease, age, and number of received vaccine doses

Suppl. Fig. 3. Vaccine-induced T cell responses are synchronized with antibody titers and display an inflammatory immune signature.

Suppl. Fig. 4. T_{helper} subsets and memory phenotypes of Omicron-reactive T cells before and after booster dose.

Suppl. Fig. 5. Heterogeneity of Omicron spike-reactive $CD8^{+}$ T_{EMRA} T cells.

Suppl. Fig. 6. Wu-Hu.1 and Omicron spike-specific responses in infected and non-infected individuals across all patient groups.

Supplementary Table S1: Cohort characteristics at baseline, vaccine doses and sampling information

Supplementary Table S2: Clinical parameters and demographics of Omicron-wave infected and non-infected individuals

Supplementary Table S3. SARS-CoV-2 spike T cell epitopes previously reported immunodominant and from natural infection

Supplementary Table S4. SARS-CoV-2 T cell epitopes previously reported immunogenic and induced by vaccination

Supplementary Table S5. Omicron variant (B.1.1.529) T cell epitopes

Supplementary Table S6: Antibody list

Supplementary Table S7: Raw data

SUPPLEMENTARY MATERIALS

SUPPLEMENTARY FIGURES

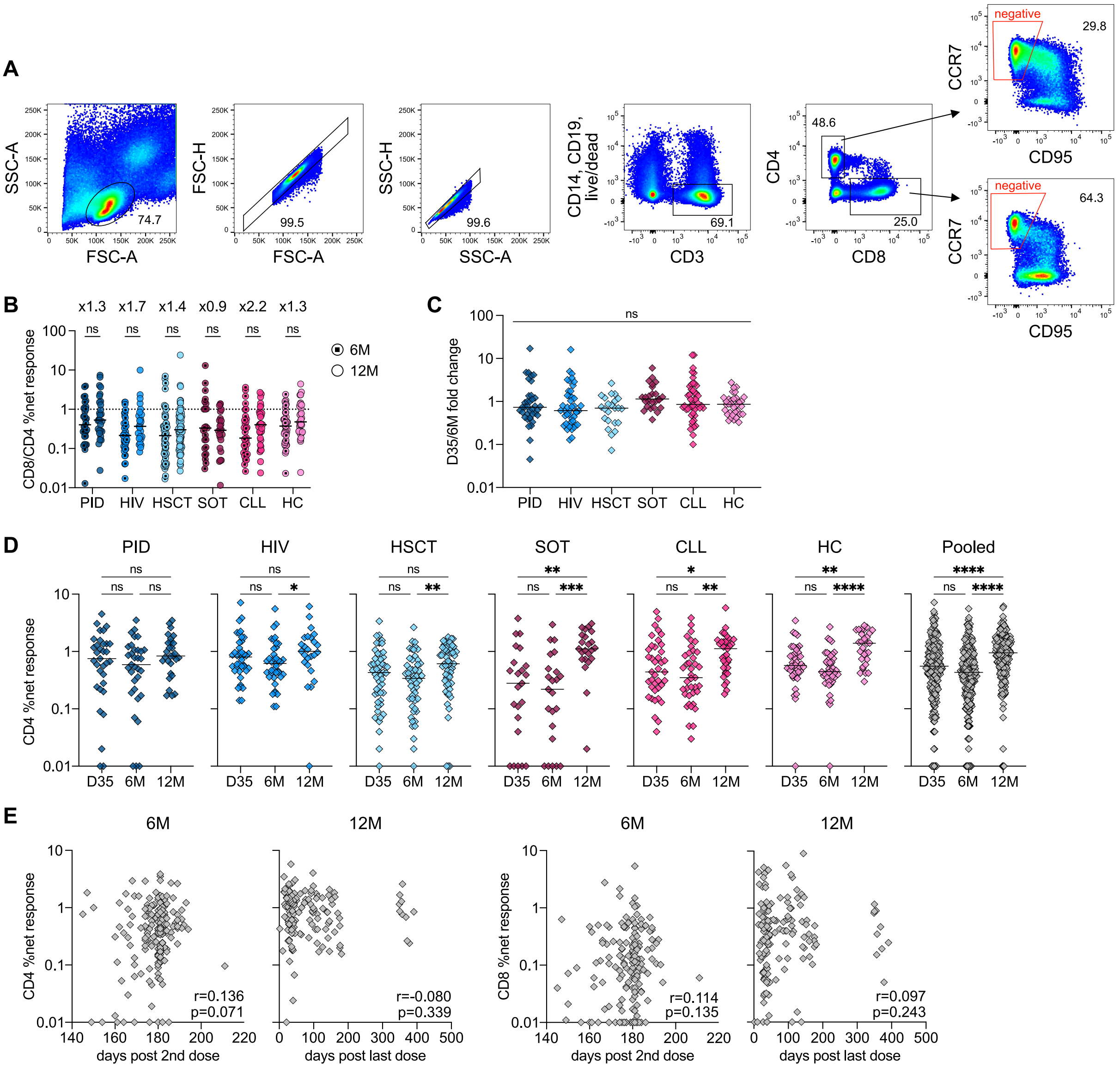

Suppl. Fig. 1. T cell responses to ancestral mRNA vaccine doses over time. (A) Gating on non-naïve/memory CD4$^+$ and CD8$^+$ T cells. Numbers indicate the percentage of the previous gate. **(B)** Comparison of pre- and post-booster CD8$^+$ to CD4$^+$ net-frequency ratio in response to Omicron full spike with indicated fold changes. **(C)** D35/6M fold change of CD4$^+$ %net responses to Wu-Hu.1 full spike calculated with data from *(18)*. **(D)** D35/6M decay rate was used to extrapolate D35 CD4$^+$ %net responses to Wu-Hu.1 full spike based on the here reported data. **(E)** Correlation of elapsed time between second or last vaccine dose with T cell %net responses to WT full spike at 6M and 12M with indicated Spearman correlation and p-values. Individuals who experienced an additional infection were excluded from this analysis. (B to E) Each dot represents one donor and lines depict the median. (B) Mann-Whitney test with Holm-Šidák posttest. (C, D) Kruskal Wallis test with Dunn's posttest. ****p <0.0001, ***p <0.001, **p <0.01, *p <0.05

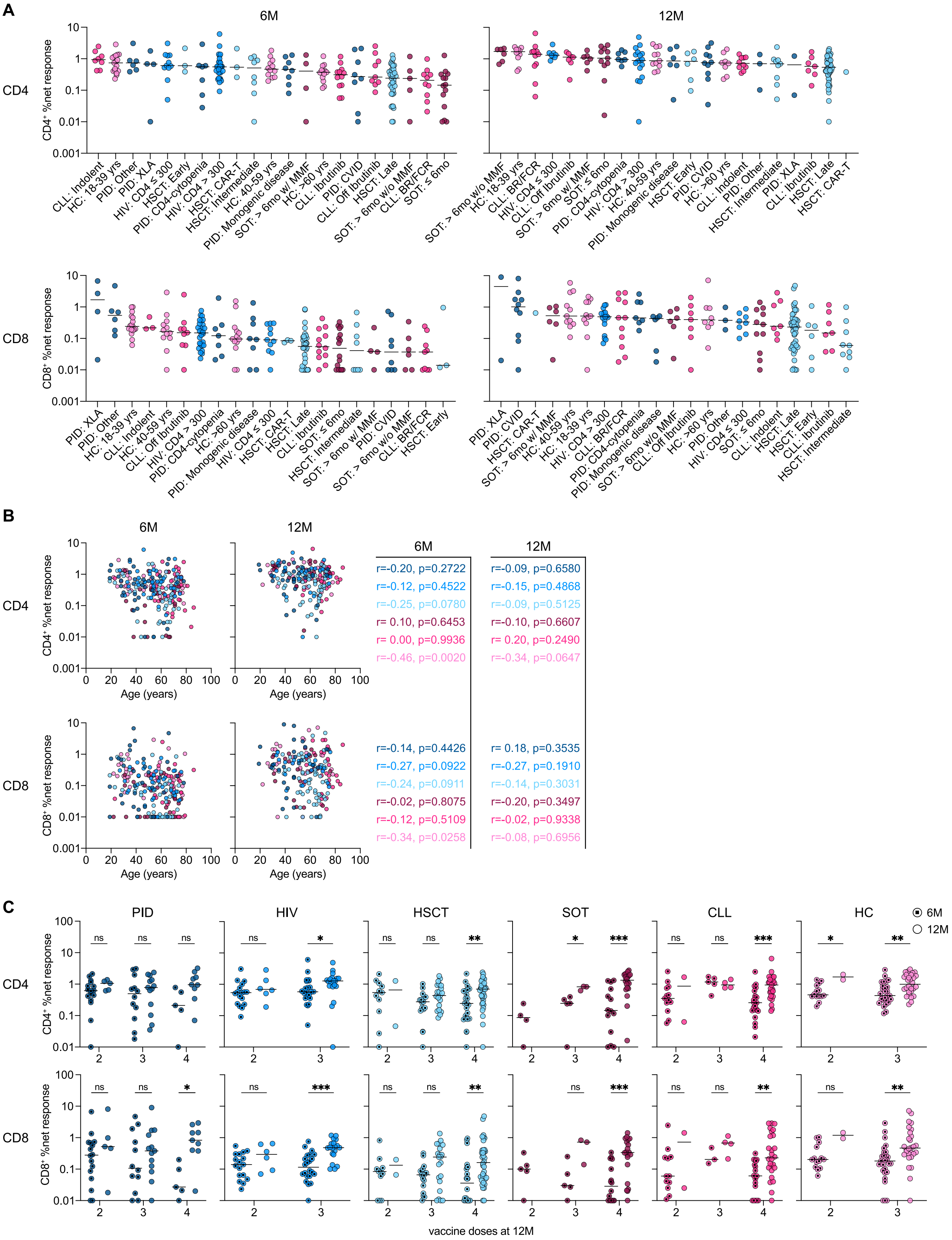

Suppl. Fig. 2. Individual variability of vaccine-induced Omicron-reactive T cell responses based on disease, age, and number of received vaccine doses. (A) Net frequencies of T cell responses across all patient subgroups. **(B)** Correlation of patient age with respective T cell responses at 6M and 12M with indicated Spearman correlation and p-values. **(C)** Comparison of pre- and post-booster T cell responses based on the number of received vaccine doses at 12M. (A to C) T cell responses to Omicron full spike peptide pool. Each dot represents one donor and lines depict the median. (C) Mann-Whitney test with Holm-Šidák posttest. ***p <0.001, **p <0.01, *p <0.05

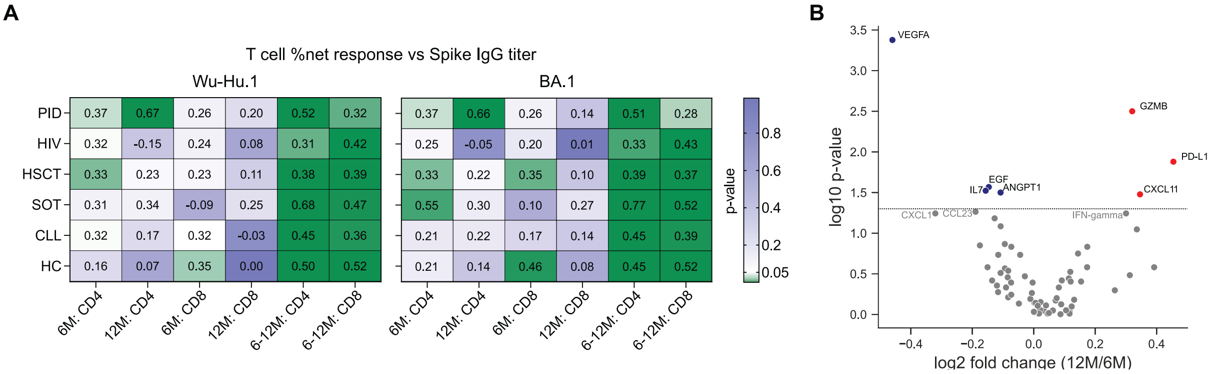

Suppl. Fig. 3. Vaccine-induced T cell responses are synchronized with antibody titers and display an inflammatory immune signature. (A) Correlation matrix of anti-Wu-Hu.1 and -Omicron BA.1 spike IgG with CD4$^+$ and CD8$^+$ T cell responses to Wu-Hu.1 full spike and Omicron full spike peptide pools, respectively, with indicated Spearman r (numbers) and p-values (color-code). **(B)** Volcano plot of differentially secreted proteins between 6M and 12M time points pooled across all patient groups. Significantly changed proteins are marked with color. Borderline non-significant proteins are visualized with names (n=30, equally distributed across groups).

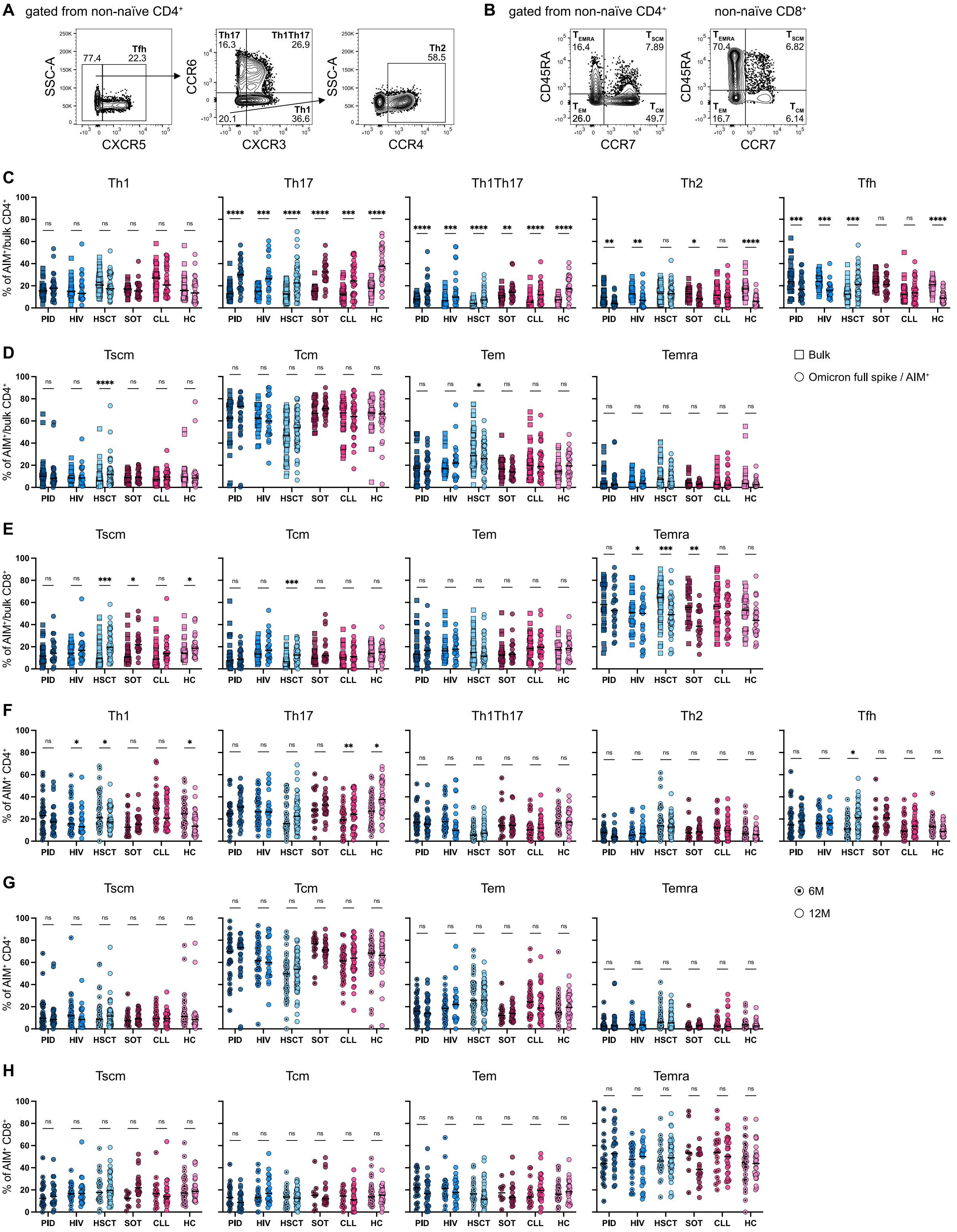

Suppl. Fig. 4. T_{helper} subsets and memory phenotypes of Omicron-reactive T cells before and after booster dose. **(A)** Gating on T-helper subsets starting from non-naïve (i.e. non-CCR7⁺CD95⁻, see Suppl. Fig. 1A) CD4⁺ T cells. **(B)** Gating on memory subsets starting from non-naïve CD4⁺ and CD8⁺ T cells. **(C)** Frequencies of indicated T_{helper} subsets of Omicron-reactive T cells and CD4⁺ bulk memory T cells at 12M time point. **(D)** Frequencies of indicated memory phenotypes of Omicron-reactive CD4⁺ T cells and CD4⁺ bulk memory T cells at 12M time point. **(E)** Frequencies of indicated memory phenotypes of Omicron-reactive CD8⁺ T cells and CD8⁺ bulk memory T cells at 12M time point. **(F)** Frequencies of indicated T_{helper} subsets of Omicron-reactive T cells before and after booster dose. **(G)** Frequencies of indicated memory phenotypes of Omicron-reactive CD4⁺ T cells before and after

booster dose. **(H)** Frequencies of indicated memory phenotypes of Omicron-reactive $CD8^+$ T cells before and after booster dose. (A, B) Numbers indicate the percentage of the previous gate. (C-G) Each dot represents one donor and lines depict the median. Wilcoxon matched-pairs signed rank test with Holm-Šidák posttest. ****p <0.0001, ***p <0.001, **p <0.01, *p <0.05.

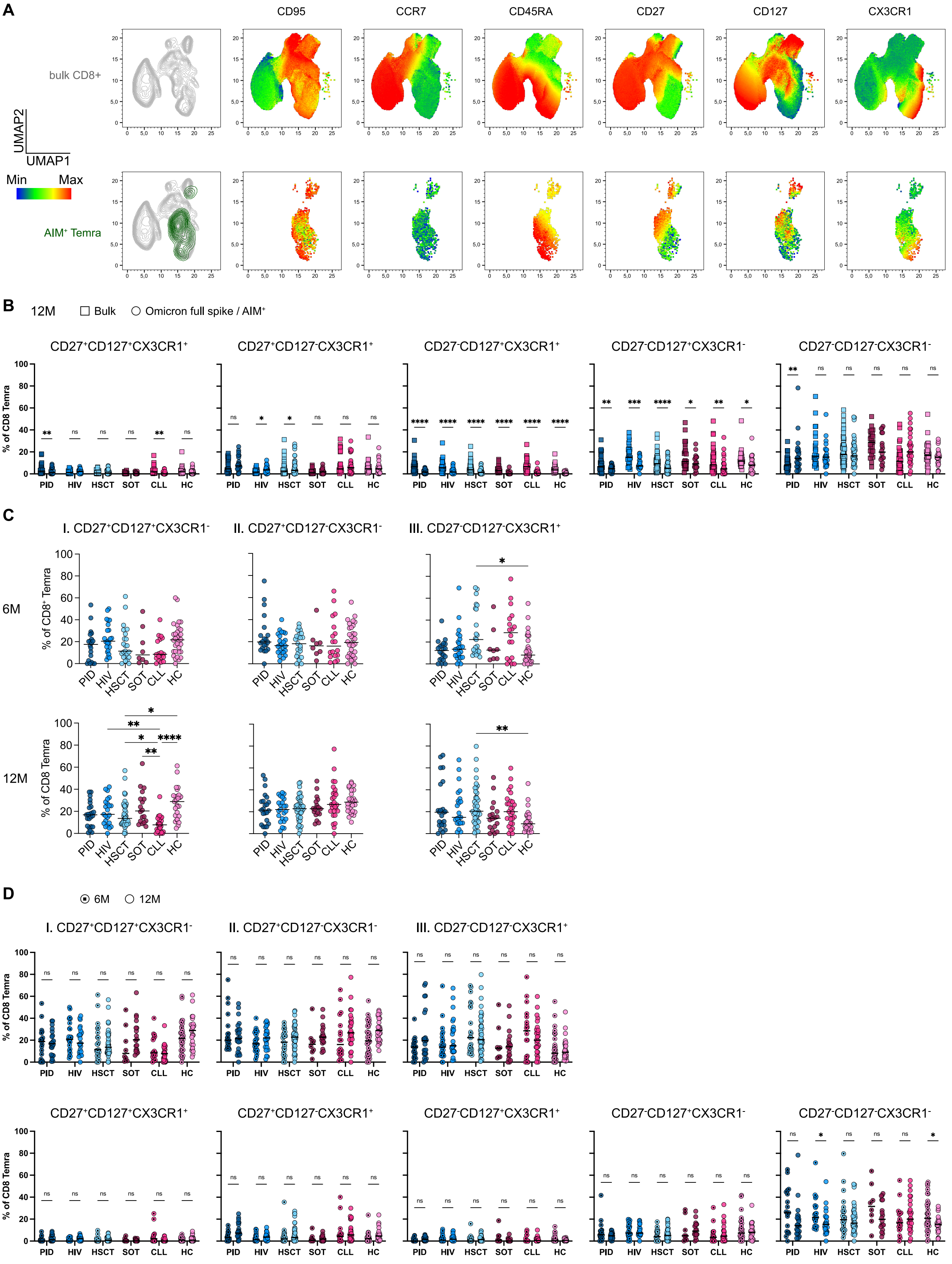

Suppl. Fig. 5. Heterogeneity of Omicron spike-reactive CD8⁺ T_{EMRA} T cells. (A) UMAP and heatmap visualization of differentiation markers of bulk CD8⁺ memory T cells (upper row) and spike-reactive CD8⁺ T_{EMRA} T cells (lower row). **(B)** Frequencies of indicated Boolean gates of Omicron spike-reactive and bulk CD8⁺ T_{EMRA} cells based on the expression of CD27, CD127, and CX3CR1. Complementary data to Fig. 4B. **(C)** Comparison of frequencies of Omicron spike-reactive CD8⁺ T_{EMRA} subsets I-III between patient groups at 6M and 12M. **(D)** Frequencies of indicated Boolean gates of Omicron spike-reactive CD8⁺ T cells based on the expression of CD27, CD127, and CX3CR1 before and after booster dose. (B to D) Each dot represents one donor, and lines depict the median. (B,

D) Wilcoxon matched-pairs signed rank test with Holm-Šidák posttest. (C) Kruskal-Wallis with Dunn's posttest. ****p <0.0001, ***p <0.001, **p <0.01, *p <0.05.

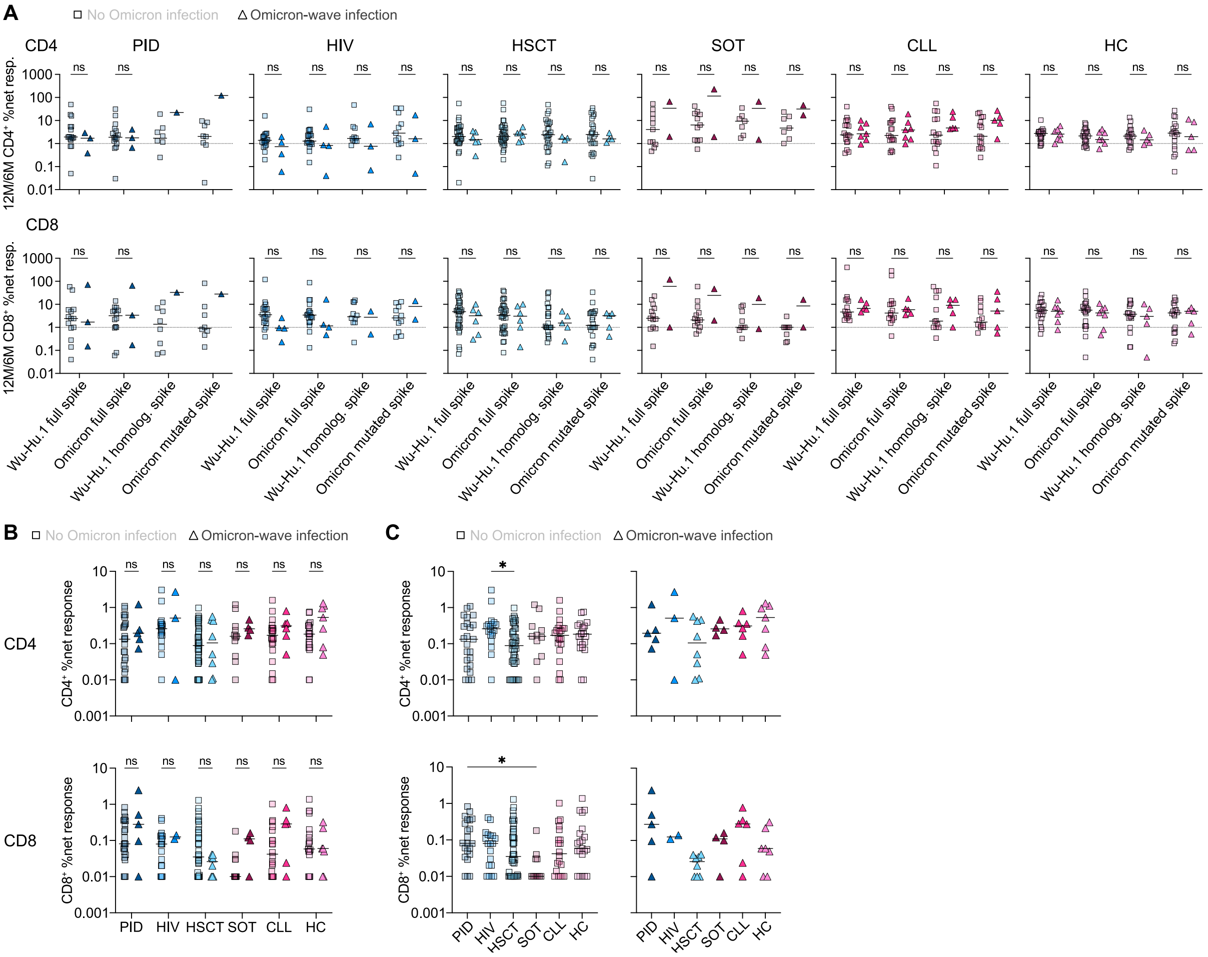

Suppl. Fig. 6. Wu-Hu.1 and Omicron spike-specific responses in infected and non-infected individuals across all patient groups. (A) Ratio of 12M/6M T cell responses to Wu-Hu.1 and Omicron spike peptide pools in Omicron-wave infected and non-infected individuals. **(B)** Net frequencies of T cell responses to the Omicron-mutated spike peptide pool in Omicron-wave infected and non-infected individuals across all patient groups. **(C)** Comparison of T cell response magnitudes to the Omicron-mutated spike peptide pool between patient groups in Omicron-wave infected and non-infected individuals. (A to C) Each dot represents one donor and lines depict the median. (A, B) Mann-Whitney test with Holm-Šidák posttest. (C) Kruskal Wallis test with Dunn`s posttest. *p <0.05.

SUPPLEMENTARY TABLES

Supplementary Table S1: Cohort characteristics at baseline, vaccine doses and sampling information

	time point	n	Female Sex	Age<br>median (IQR)	Vaccine doses	Days between last dose and sampling<br>median (IQR)	Subgroups
**PID**	**6M**	33	48.5%	41.0 (32.5–53.5)	2 (33/33)	176.0 (162.0–183.0)	CVID (8/33)<br>XLA (4/33)<br>CD4-cytopenia (7/33)<br>Monogenic disease (8/33)<br>Other (6/33)
	**12M**	28	50.0%	47.0 (39.0–55.5)	1 (1/28)<br>2 (5/28)<br>3 (13/28)<br>4 (9/28)	93.5 (36.5–139.8)	CVID (10/28)<br>XLA (2/28)<br>CD4-cytopenia (7/28)<br>Monogenic disease (6/28)<br>Other (3/28)
**HIV**	**6M**	39	43.6%	54.0 (46.0–64.0)	2 (33/33)	181.0 (180.0–183.0)	CD4 ≤300 (11/39)<br>CD4 >300 (28/39)
	**12M**	25	48.0%	56.0 (40.5–66.0)	2 (6/25)<br>3 (19/25)	101.5 (67.0–151.8)	CD4 ≤300 (8/25)<br>CD4 >300 (17/25)
**HSCT**	**6M**	51	41.2%	59.0 (50.0–67.0)	1 (1/51)<br>2 (50/51)	179.5 (176.3–180.0)	CAR-T (3/51)<br>Early (3/51)<br>Intermediate (8/51)<br>Late (37/51)
	**12M**	59	47.5%	62.0 (53.0–68.0)	1 (1/59)<br>2 (1/59)<br>3 (21/59)<br>4 (36/59)	41.0 (29.0–152.3)	CAR-T (1/59)<br>Early (6/59)<br>Intermediate (9/59)<br>Late (43/59)
**SOT**	**6M**	23	43.5%	57.0 (47.0–67.0)	2 (33/33)	175.0 (167.8–177.0)	≤6mo (14/23)<br>>6mo w/ MMF (4/23)<br>>6mo w/o MMF (5/23)
	**12M**	23	56.5%	52.0 (40.0–63.0)	3 (3/23)<br>4 (20/23)	37.0 (26.0–42.8)	≤6mo (11/23)<br>>6mo w/ MMF (6/23)<br>>6mo w/o MMF (6/23)
**CLL**	**6M**	39	33.3%	70.0 (63.0–75.0)	2 (33/33)	181.0 (179.0–182.0)	BR/FCR (10/39)<br>Ibrutinib (12/39)<br>Indolent (8/39)<br>Off Ibrutinib (9/39)
	**12M**	35	34.3%	71.0 (63.0–75.0)	2 (2/35)<br>3 (4/35)<br>4 (29/35)	34.0 (22.0–39.0)	BR/FCR (11/35)<br>Ibrutinib (7/35)<br>Indolent (10/35)<br>Off Ibrutinib (7/35)
**HC**	**6M**	42	54.8%	52.0 (32.8–68.3)	1 (2/42)<br>2 (40/42)	182.0 (181.0–185.0)	18-39 yrs (17/42)<br>40-59 yrs (12/42)<br>>60 yrs (13/42)
	**12M**	30	53.3%	53.0 (33.0–66.5)	2 (2/30)<br>3 (28/30)	105.0 (93.0–138.5)	18-39 yrs (11/30)<br>40-59 yrs (11/30)<br>>60 yrs (8/30)

Abbreviations: n: number, IQR: interquartile range, PID: primary immunodeficiency disorders, HIV: human immunodeficiency virus type 1, HSCT: hematopoietic stem cell transplantation, SOT: solid organ transplantation, CLL: chronic lymphocytic leukemia, HC: healthy controls, 6M: 6 month, 12M: 12 month, CVID: common variable immunodeficiency, XLA: X-linked agammaglobulinemia, CAR-T: chimeric antigen receptor T cell therapy, mo: months, w/: with, w/o: without, MMF: mycophenolate mofetil, BR/FCR: previous treatment with bendamustine and rituximab / fludarabine, cyclophosphamide and rituximab

Supplementary Table S2: Clinical parameters and demographics of Omicron-wave infected and non-infected individuals

	Omicron wave	n	Female Sex	Age median (IQR)	Vaccine doses at 12M	Disease score (WHO, 1-10)	Subgroups
**PID**	**non-infected**	23	60.9%	47.0 (39.0–59.0)	1 (1/23)<br>2 (4/23)<br>3 (11/23)<br>4 (7/23)		CVID (7/23)<br>XLA (1/23)<br>CD4-cytopenia (6/23)<br>Monogenic disease (6/23)<br>Other (3/23)
	**infected**	5	0.0%	47.0 (37.0–50.5)	2 (1/5)<br>3 (2/5)<br>4 (2/5)	1 (3/5)<br>2 (2/5)	CVID (3/5)<br>XLA (1/5)<br>CD4-cytopenia (1/5)<br>Monogenic disease (0/5)<br>Other (0/5)
**HIV**	**non-infected**	21	52.4%	60.0 (40.5–69.0)	2 (6/21)<br>3 (15/21)		CD4 ≤300 (7/21)<br>CD4 >300 (14/21)
	**infected**	4	0.0%	50.0 (37.0–55.5)	3 (4/4)	1 (4/4)	CD4 ≤300 (1/4)<br>CD4 >300 (3/4)
**HSCT**	**non-infected**	51	47.1%	60.0 (52.0–67.0)	1 (1/51)<br>3 (16/51)<br>4 (34/51)		CAR-T (1/51)<br>Early (4/51)<br>Intermediate (8/51)<br>Late (38/51)
	**infected**	8	50.0%	68.0 (62.0–70.3)	2 (1/8)<br>3 (5/8)<br>4 (2/8)	1 (7/8)<br>5 (1/8)	CAR-T (0/8)<br>Early (2/8)<br>Intermediate (1/8)<br>Late (5/8)
**SOT**	**non-infected**	17	58.8%	50.0 (37.5–63.0)	3 (2/17)<br>4 (15/17)		≤6mo (8/17)<br>>6mo w/ MMF (4/17)<br>>6mo w/o MMF (5/17)
	**infected**	6	50.0%	56.5 (37.5–63.3)	3 (1/6)<br>4 (5/6)	1 (4/6)<br>3 (2/6)	≤6mo (3/6)<br>>6mo w/ MMF (2/6)<br>>6mo w/o MMF (1/6)
**CLL**	**non-infected**	27	33.3%	70.0 (62.0–74.0)	2 (1/27)<br>3 (2/27)<br>4 (24/27)		BR/FCR (10/27)<br>Ibrutinib (5/27)<br>Indolent (7/27)<br>Off Ibrutinib (5/27)
	**infected**	8	37.5%	74.5 (65.5–81.8)	2 (1/8)<br>3 (2/8)<br>4 (5/8)	1 (7/8)<br>2 (1/8)	BR/FCR (1/8)<br>Ibrutinib (2/8)<br>Indolent (3/8)<br>Off Ibrutinib (2/8)
**HC**	**non-infected**	23	52.2%	56.0 (36.0–69.0)	2 (1/23)<br>3 (22/23)		18-39 yrs (6/23)<br>40-59 yrs (9/23)<br>>60 yrs (8/23)
	**infected**	7	57.1%	33.0 (30.0–55.0)	2 (1/7)<br>3 (6/7)	1 (7/7)	18-39 yrs (5/7)<br>40-59 yrs (2/7)<br>>60 yrs (0/7)

Abbreviations: n: number, IQR: interquartile range, PID: primary immunodeficiency disorders, HIV: human immunodeficiency virus type 1, HSCT: hematopoietic stem cell transplantation, SOT: solid organ transplantation, CLL: chronic lymphocytic leukemia, HC: healthy controls, 6M: 6 month, 12M: 12 month, CVID: common variable immunodeficiency, XLA: X-linked agammaglobulinemia, CAR-T: chimeric antigen receptor T cell therapy, mo: months, w/: with, w/o: without, MMF: mycophenolate mofetil, BR/FCR: previous treatment with bendamustine and rituximab / fludarabine, cyclophosphamide and rituximab

Supplementary Table S3: SARS-CoV-2 spike T cell epitopes previously reported immunodominant and from natural infection

No.	HLA	Peptide
1	A01:01	WTAGAAAYY
2	A01:01	LTDEMIAQY
3	A01:01	CNDPFLGVYY
4	A01:01	FCNDPFLGVY
5	A01:01	PLLTDEMIAQY
6	A01:01	QTLLALHRSY
7	A01:01	SGWTAGAAAYY
8	A01:01	SSANNCTFEY
9	A01:01	STECSNLLL
10	A01:01	TDEMIAQY
11	A01:01	TTEILPVSM
12	A02:01	NLNESLIDL
13	A02:01	RLDKVEAEVQI
14	A02:01	RLITGRLQSL
15	A02:01	VLSFELLHA
16	A02:01	VLYQDVNCTEV
17	A02:01	VTWFHAIHV
18	A02:01	YLQPRTFLL
19	A02:01	RLNEVAKNL
20	A02:01	YQDVNCTEV
21	A02:01	ALNTLVKQL
22	A02:01	HLMSFPQSA
23	A03:01	GVYFASTEK
24	A03:01	GVYYPDKVFR
25	A03:01	KCYGVSPTK
26	A24:02	QPYRVVVLSF
27	A24:02	RVYSSANNCTF
28	A24:02	TQDLFLPFF
29	A24:02	VYSTGSNVF
30	A24:02	YFPLQSYGF
31	A24:02	GYQPYRVVV
32	A24:02	NYNYLYRLF
33	A24:02	QYIKWPWYI
34	A24:02	YYHKNNKSW

35	A24:02	EYVSQPFLM
36	A24:02	IYKTPPIKDF
37	A24:02	YYVGYLQPRTF
38	A24:02	TVYDPLQPEL
39	A24:02	YLQPRTFLL
40	A24:02	GNYNYLYRLF
41	A24:02	GYLQPRTF
42	A24:02	LFLPFFSNVTW
43	A24:02	PFAMQMAYRF
44	A24:02	PFGEVFNATRF
45	A24:02	SFKEELDKYF
46	A24:02	VFVSNGTHW
47	A24:02	VGYLQPRTFL
48	B07:02	SPRRARSV
49	B07:02	VVNQNAQAL
50	B07:02	APGQTGKIA
51	B07:02	APHGVVFL
52	B07:02	APHGVVFLHV
53	B07:02	KPSKRSFIEDL
54	B08:01	LPQGFSAL
55	B08:01	QPYRVVVL
56	B08:01	FKNLREFVF
57	B08:01	DLPQGFSAL
58	B08:01	LITGRLQSL
60	B08:01	VFQTRAGCL
59	B08:01	VAKNLNESL
61	B15:01	CVADYSVLY
62	B15:01	GQTGKIADY
63	B15:01	GQTGKIADYNY
64	B35:01	HADQLTPTW
65	B35:01	LPFNDGVYF
66	B35:01	VLPFNDGVYF

Supplementary Table S4: SARS-CoV-2 T cell epitopes previously reported immunogenic and induced by vaccination

No.	HLA	Peptide
1	A01:01	FTSDYYQLY

2	A01:01	FVFKNIDGY
3	A01:01	TILDGISQY
4	A01:01	TPSGTWLTY
5	A01:01	CTDDNALAYY
6	A01:01	CTEIDPKLDNY
7	A01:01	FTCASEYTGNY
8	A01:01	PTDNYITTY
9	A01:01	STECSNLLLQY
10	A01:01	TTDPSFLGRY
11	A01:01	YFTSDYYQLY
12	A02:01	NLLLLFVTV
13	A02:01	SIWNLDYIINL
14	A02:01	SLDTYPSLETI
15	A02:01	SLIDLQEL
16	A02:01	SLINTLNDL
17	A02:01	SLLSVLLSM
18	A02:01	SMWSFNPET
19	A02:01	TLIVNSVLL
20	A02:01	YLDGADVTKI
21	A02:01	YLNTLTLAV
22	A02:01	YLVQQESPFV
23	A02:01	VLQLPQGTTL
24	A02:01	KVDGVDVEL
25	A02:01	VLNDILSRL
26	A02:01	YLDAYNMMI
27	A02:01	ALWEIQQV
28	A02:01	AVASKILGL
29	A02:01	FLAFVVFLL
30	A02:01	HLVDFQVTIA
31	A02:01	KLKDCVMYA
32	A02:01	KLPDDFTGCV
33	A02:01	KLWAQCVQL
34	A02:01	LLLLDRLNQL
35	A03:01	KLFDRYFKY
36	A03:01	KTFPPTEPK
37	A03:01	KTFPPTEPKK
38	A03:01	KTIQPRVEK

39	A03:01	QVVNVVTTK
40	A03:01	RASANLAATK
41	A03:01	VTNNTFTLK
42	A24:02	SYATHSDKF
43	A24:02	FYAYLRKHF
44	A24:02	QYIKWPWYI
45	A24:02	SYFTSDYYQLY
46	A24:02	PFVVSTGYHF
47	A24:02	SWMESEFRV
48	A24:02	VYRGTTTYKL
49	B07:02	EPVLKGVKL
50	B07:02	LPQGFSAL
51	B07:02	MIAQYTSAL
52	B07:02	TPINLVRDL
53	B07:02	SPRRARSVA
54	B07:02	TPCSFGGVSV
55	B08:01	TLDSKTQSL
56	B08:01	MIAQYTSAL

Supplementary Table S5: Omicron variant (B.1.1.529) T cell epitopes

No.	HLA	Peptide
1	HLA-A01:01	QTGNIADYNY
2	HLA-A01:01	KLDSKVSGNY
3	HLA-A01:01	VSGNYNYLY
4	HLA-A01:01	GAEYVNNSY
5	HLA-A01:01	KSYGFQPTY
6	HLA-A01:01	RSDVLLPFTQY
7	HLA-A01:01	CNDPFLDHK
8	HLA-A01:01	FCNDPFLDHKN
9	HLA-A01:01	LAPFFTFKCY
10	HLA-A01:01	LAPFFAFKCY
11	HLA-A02:01	NIADYNYKL
12	HLA-A02:01	VLYQGVNCTEV
13	HLA-A02:01	KLQDVVNHNA
14	HLA-A02:01	FLARGVVFM
15	HLA-A02:01	KLLEEWNLV

16	HLA-A02:01	SVLNDIFSRL
17	HLA-A02:01	VLNDIFSRL
18	HLA-A02:01	WLDMVDTSF
19	HLA-A02:01	LLWPVTLTC
20	HLA-A03:01	HVISGTNGTK
21	HLA-A03:01	GVYFASIEK
22	HLA-A03:01	NLAPFFTFK
23	HLA-A03:01	TLVKQLSSK
24	HLA-A03:01	KSYGFQPTY
25	HLA-A24:02	IYSKHTPII
26	HLA-A24:02	LYNLAPFFTF
27	HLA-A24:02	IYKTPPIKYF
28	HLA-A24:02	KYFGGFNF
29	HLA-A24:02	PFDEVFNATRF
30	HLA-A24:02	LYQPPQISI
31	HLA-A24:02	QGYKSVNIIF
32	HLA-A24:02	YFPLRSYSF
33	HLA-A24:02	YFPLRSYGF
34	HLA-B07:01	TPIIVRDLP
35	HLA-B07:01	TPIIVREPE
36	HLA-B07:01	VAKSHNITL
37	HLA-B08:01	HQPYRVVVL
38	HLA-B08:01	AQKFKGLTV

Supplementary Table S6: Antibody list

**Antibody (clone)**	**Catolog number**	**Source**	**RRID**
CD3–BUV805 (UCHT1)	612895	BD Biosciences	AB_2870183
CCR6–BUV737 (11A9)	612780	BD Biosciences	AB_2870109
CD4–BUV496 (SK3)	612936	BD Biosciences	AB_2870220
CD8–BUV395 (RPA-T8)	563795	BD Biosciences	AB_2722501
CD27-BV786 (O323)	302832	Biolegend	AB_2562674
CD69-BV650 (FN50)	310934	Biolegend	AB_2563158
CD45RA–BV570 (HI100)	304132	BioLegend	AB_2563813
CD14–BV510 (M5E2)	301842	BioLegend	AB_2561946
CD19–BV510 (HIB19)	302242	BioLegend	AB_2561668
CD154–BV421 (24-31)	310824	BioLegend	AB_2562721

CCR4–BB700 (1G1)	566475	BD Biosciences	AB_2744302
CXCR5–BB515 (RF8B2)	564624	BD Biosciences	AB_2738871
CD137–PE-Cy7 (4B4-1)	309818	BioLegend	AB_2207741
CD127-PE/Cyanine5 (A019D5)	351324	BioLegend	AB_10915554
CD95-PE/Dazzle™ 594 (DX2)	305634	BioLegend	AB_2564221
CX3CR1-PE (2A9-1)	341604	BioLegend	AB_1595456
CX3CR1-BUV661 (2A9-1)	750690	BD Biosciences	AB_2874813
CCR7–APC-Cy7 (G043H7)	353212	BioLegend	AB_10916390
CXCR3-AF647 (G025H7)	353712	BioLegend	AB_10962948
CD57-Pacific Blue (HNK-1)	359608	BioLegend	AB_2562459
Granzyme B-BB790 (GB11)	624296	BD Biosciences	Custom
Granzyme K-PerCP-eFluor710 (G3H69)	46-8897-42	ThermoFisher	AB_2573854
TCF1-AF488 (C63D9)	6444S	Cell Signaling	n/a
KLRG1-PE (REA261)	130-120-566	Miltenyi	AB_2784406
